# Fusion of FokI and catalytically inactive prokaryotic Argonautes enables site-specific programmable DNA cleavage

**DOI:** 10.1016/j.jbc.2024.107720

**Published:** 2024-08-28

**Authors:** Qiaochu Wang, Gundra Sivakrishna Rao, Tin Marsic, Rashid Aman, Magdy Mahfouz

**Affiliations:** Laboratory for Genome Engineering and Synthetic Biology, Division of Biological Sciences, 4700 King Abdullah University of Science and Technology, Saudi Arabia

**Keywords:** genome editing, Prokaryotic Argonautes, catalytically inactive Argonautes, FokI nuclease, peptide nucleic acids, double strand DNA break

## Abstract

Site-specific nucleases are crucial for genome engineering applications in medicine and agriculture. The ideal site-specific nucleases are easily reprogrammable, highly specific in target site recognition, and robust in nuclease activities. Prokaryotic Argonaute (pAgo) proteins have received much attention as biotechnological tools due to their ability to recognize specific target sequences without a protospacer adjacent motif, but their lack of intrinsic dsDNA unwinding activity limits their utility in key applications such as gene editing. Recently, we developed a pAgo-based system for site-specific DNA cleavage at physiological temperatures independently of the DNA form, using peptide nucleic acids (PNAs) to facilitate unwinding dsDNA targets. Here, we fused catalytically dead pAgos with the nuclease domain of the restriction endonuclease FokI and named this modified platform PNA-assisted FokI-(d)pAgo (PNFP) editors. In the PNFP system, catalytically inactive pAgo recognizes and binds to a specific target DNA sequence based on a programmable guide DNA sequence; upon binding to the target site, the FokI domains dimerize and introduce precise dsDNA breaks. We explored key parameters of the PNFP system including the requirements of PNA and guide DNAs, the specificity of PNA and guide DNA on target cleavage, the optimal concentration of different components, reaction time for invasion and cleavage, and ideal temperature and reaction buffer, to ensure efficient DNA editing *in vitro*. The results demonstrated robust site-specific target cleavage by PNFP system at optimal conditions *in vitro*. We envision that the PNFP system will provide higher editing efficiency and specificity with fewer off-target effects *in vivo*.

Genome-editing technologies enable site-specific manipulation of target DNA sequences and have promising applications in fundamental research, gene therapy, molecular diagnostics, and biotechnology. Programmable nucleases are key components of many genome-editing technologies, as they recognize specific, user-selected target sites and introduce double-stranded breaks (DSBs) in the target DNA (1). Changes in the DNA sequence are then accomplished *via* the cellular DNA repair machinery, by either nonhomologous end-joining or homology-directed repair pathways (1). To date, various programmable nucleases have been established for laboratory and clinical use, including meganucleases (2), zinc finger nucleases (ZFNs) (3), transcription activator-like effector nucleases (TALENs) (4, 5), and clustered regularly interspaced short palindromic repeats and CRISPR-associated nuclease (CRISPR/Cas) systems (6).

ZFNs and TALENs are chimeric nucleases created by fusing sequence-specific DNA-binding protein domains with the nuclease domain of the restriction endonuclease FokI from *Flavobacterium okeanokoites*. In these chimeric nucleases, the DNA-binding domain recognizes the target sequence and the FokI nuclease domain forms dimers that cleave dsDNA at the target site (7, 8). The subdomains responsible for target recognition and target cleavage in these chimeric nucleases function independently; therefore, these platforms can be reprogramed to act on various targets (9, 10, 11). However, reprogramming the target sequence involves changing the protein sequence of the nuclease, which employs protein–DNA interactions for DNA recognition. By contrast, CRISPR/Cas systems rely on Watson-Crick base paring between their single guide RNAs and the target DNA sequence, providing greater simplicity and flexibility in reprogramming for targeting any site of interest (1). CRISPR/Cas systems have revolutionized genome editing and have broad utility in molecular biological research and in therapeutics, inspiring the discovery of myriad Cas protein variants and the development of CRISPR-based biotechnological platforms (12). For example, Guilinger *et al.* developed a chimeric nuclease by fusing a catalytically inactive Cas9 nuclease with the nuclease domain of FokI, termed fCas9, to achieve better targeting specificity and fewer off-target effects (13). Despite their broad utility, CRISPR/Cas systems have a few limitations, such as the large size of the Cas protein and the requirement for a protospacer adjacent motif (PAM) adjacent to the target site.

Prokaryotic Argonautes (pAgos) recognize their targets based on a guide oligonucleotide and allow PAM-independent DNA targeting (14). Therefore, pAgos may have potential uses in programmable nucleases. Argonaute proteins belong to a diverse family of oligonucleotide-guided nucleases, first discovered in eukaryotes, that have since been reported in all domains of life (15, 16, 17). The pAgos have diverse domain architectures and can be divided into long pAgos, short pAgos, and PIWI-RE proteins (P-element-induced wimpy testis [PIWI] with conserved R [Arg] and E [Glu] residues) (18). Long pAgos show high sequence similarity to eukaryotic Agos as both types contain N-terminal, PIWI-Argonaute-Zwille (PAZ), middle (MID), and PIWI domains. The MID and PAZ domains are responsible for loading of the guide oligonucleotide by interacting with the 5′ and 3′ ends of the oligonucleotide guide, respectively (19, 20, 21).

The endonuclease activity of pAgos relies on the presence of an intact PIWI domain that possesses an RNase H-like fold with a conserved DEDX (where X denotes aspartic acid, histidine, or lysine) catalytic motif (22). An incomplete PIWI motif and substitutions of the amino acid residues within the PIWI domain present in many Argonaute proteins renders them incapable of cleaving the target, although they retain target recognition and binding activity (15, 16, 17). Since a single pAgo molecule contains a single cleavage domain, a pair of pAgos that nick opposite strands simultaneously is required to generate DSBs (23).

Because pAgos enable site-specific DNA cleavage in a guide-dependent manner, they are promising tools for developing programmable DNA editors. Moreover, the discovery of pAgos from mesophilic species like *Clostridium butyricum*, *Limnothrix rosea*, and *Kurthia massiliensis* expands the range of reaction temperatures for potential *in vivo* applications (24, 25, 26). However, one major challenge in harnessing pAgos for gene editing is their lack of intrinsic dsDNA unwinding activity (18), limiting their application on unwound dsDNA structures, usually present at high temperature, regions with low GC content in supercoiled DNA, or during cellular processes like replication and transcription (27, 28). In addition, accessory proteins, like ssDNA-binding proteins or helicases, can assist pAgo-mediated DNA cleavage *in vitro* (29, 30).

Peptide nucleic acids (PNAs) are DNA analogs, synthesized by replacing the phosphodiester backbone of DNA with peptide bond–linked N-(2-aminoethyl)-glycine units (31). Due to their neutrally charged backbone, PNAs can bind to DNA or RNA with much less electrostatic repulsion and higher binding affinity than during normal DNA–DNA, DNA–RNA, or RNA–RNA interactions, generating much more stable hybrids. PNAs have been deployed for use in genome editing due to their ability to form triplex structures with genomic DNA, mainly by forming PNA–DNA–DNA triplexes *via* Hoogsteen base pairing or by invading dsDNA through Watson-Crick base pairing (32). Site-specific gene editing accomplished by co-delivering triplex-forming gamma tail clamp PNAs (*γ*tcPNAs) and donor DNA templates *in vivo* have been described in several reports (33, 34, 35, 36, 37). The triplex structure formed by *γ*tcPNA and dsDNA is hypothesized to distort target DNA, which can then be recognized by endogenous DNA repair factors and thus provoke DNA repair and homologous recombination assisted by externally provided donor DNA templates (38, 39, 40).

Inspired by programmable nucleases like ZFNs and TALENs, we designed a DNA-editing platform by fusing a nuclease-inactive pAgo mutant ((d)pAgo) to the FokI nuclease domain, named PNA-assisted FokI-(d)pAgo editors (PNFP editors). We coupled this fusion protein with the ability of PNAs to invade dsDNA with great specificity. Each reagent in the PNFP has a designated function, such that two PNA molecules unwind the adjacent target dsDNA and acts as an “opener,” sequence specifically; two (d)pAgos with the help of corresponding guide DNAs recognize the two PNA unwounded ssDNA regions; and two FokI nucleases fused with the different (d)pAgos can be localized to the adjacent target regions. Further, cleavage is accomplished at the targeted dsDNA region by the dimerization of the FokI nuclease domain. The fusion proteins are expected to increase targeting specificity, as simultaneous recognition and binding on two adjacent sites are required for efficient DSB generation. Compared to other well-defined platforms, PNFP editors are easily reprogrammed without laborious protein engineering or molecular cloning procedures, enabling versatile targeting in a PAM-independent manner. In this work, we established these PNFP editors for target-specific DNA cleavage *in vitro* and addressed their potential as tools for applications such as precise genome engineering, genome assembly, and molecular cloning.

## Results

### Design and concept of PNFP editors

**Figure 1:**
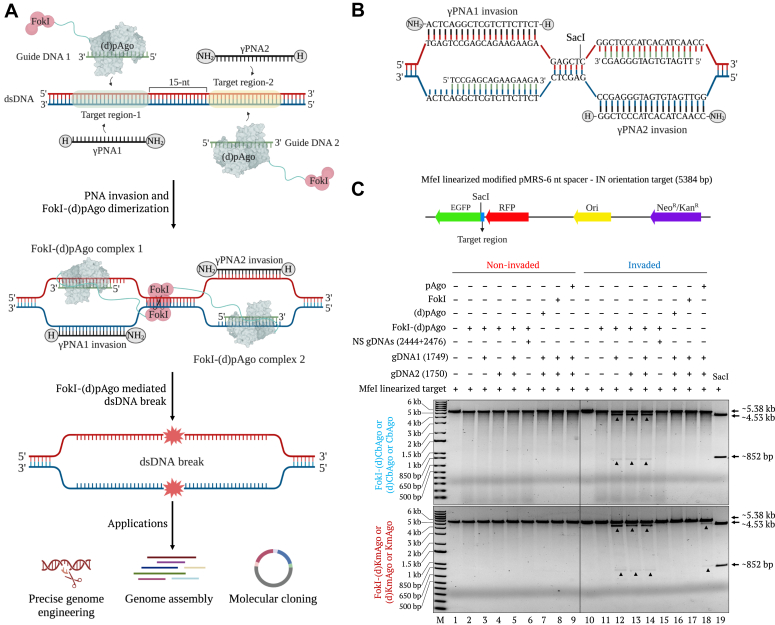
**PNA-assisted FokI-(d)pAgo cleavage of dsDNA.***A*, diagram of the PNA-assisted FokI-(d)pAgo–mediated cleavage of dsDNA. In the first step, two PNA molecules recognize and invade the adjacent target dsDNA sequences. Then, the two FokI-(d)pAgo–gDNA complexes specifically bind to the unwound ssDNA. Finally, the two FokI proteins dimerize at the spacer region between the two FokI-(d)pAgo–gDNA complexes and introduce the double-strand breaks (DSBs). *B*, sequence information and binding positions of γPNA1, γPNA2, and the two guide DNAs in the modified pMRS-6 nt spacer - IN orientation target. *C*, *top*, diagram of the *Mfe*I-linearized, modified pMRS-6 nt spacer - IN orientation target. *Bottom*, gel images showing the FokI-(d)CbAgo- (*top*) and FokI-(d)KmAgo- (*bottom*) mediated cleavage of the noninvaded (Lanes 1–6) and γPNA1&2-invaded (Lanes 10–15) target. FokI-(d)pAgo target cleavage was tested using no guides, single specific guides, a pair of specific guides, and a pair of nonspecific guides. Reactions including (d)pAgo, FokI, or intact pAgo only for cleavage in the presence of two specific guides were included as controls (lanes 7–9 [non-invaded] and 16 to 18 [γPNA1&2-invaded]). In the gel picture the FokI-(d)pAgo is either FokI-(d)CbAgo or FokI-(d)KmAgo, the (d)pAgo is either (d)CbAgo or (d)KmAgo, and the pAgo is either CbAgo or KmAgo. The *Sac*I restriction enzyme size control is shown in lane 19. Lane M, 1-kb plus DNA marker.

To generate the PNFP editors, we employed a pair of PNAs as “openers” to assist the DNA editing mediated by a guide DNA preloaded onto FokI-(d)pAgo (Fig. 1*A*). In detail, to generate the desired DSBs in DNA substrates, a pair of *γ*-modified PNA molecules (*γ*PNAs) is needed to invade two closely located target regions. The simultaneous invasion by two *γ*PNAs will result in two displaced ssDNA segments that are accessible to FokI-(d)pAgos loaded with guides complementary to the unwounded ssDNA regions. When the two guide-loaded FokI-(d)pAgos recognize and bind to their adjacent targets, the two closely positioned FokI nuclease domains dimerize and generate DSBs (Fig. 1*A*).

We constructed the FokI-(d)pAgos using mesophilic CbAgo from *C. butyricum* and KmAgo from *K. massiliensis*, previously used for PNP editors, with the “D” in the DEDX motif mutated to “A” to obtain catalytically dead versions (41) (File S1, *A* and *B* and Table S1). We then fused the resulting (d)CbAgo or (d)KmAgo to the FokI cleavage domain *via* a flexible linker (42), with a nuclear localization sequence (NLS) from simian virus 40 added to the N terminus. The FokI nuclease domain is positioned at the N terminus of each (d)pAgo (Fig. S1*A*). The purified FokI-(d)CbAgo and FokI-(d)KmAgo proteins were examined on SDS-PAGE gel showing the sizes around 114 kDa (Fig. S1*B*).

### Proof-of-concept of PNA-assisted FokI-(d)pAgo editors

For the proof-of-concept analysis, we designed and employed a pMRS plasmid containing *γ*PNA1- and *γ*PNA2-binding regions with a 6-nucleotide (nt) spacer. Accordingly, we designed two 5′ phosphorylated guide DNAs (guide DNA1 and guide DNA2) with 3′ ends facing “IN” orientation (modified pMRS-6 nt spacer - IN orientation target) (Fig. 1*B*). We performed proof-of-concept assays using FokI-(d)CbAgo and FokI-(d)KmAgo on circular and *Mfe*I-linearized dsDNA targets (noninvaded or invaded) with different combinations of guides (no guide, single guide [guide DNA1 or guide DNA2], a pair of guides, or nonspecific guides). In all cases, when we used a pair of specific guides, we obtained efficient target cleavage, as indicated by detection of bands of the expected sizes (Figs. S1*C* and 1*C*). By contrast, we saw no evidence for DSBs in reactions with noninvaded plasmids, indicating the necessity of *γ*PNA invasion. Interestingly, we also detected efficient DSB generation in the reactions using a single guide DNA, showing comparable activity to that observed with two guide DNAs. We also confirmed that the FokI and (d)pAgo subdomains are both required to generate DSBs, as we did not detect sequence-specific DNA cleavage in reactions using only the (d)pAgo or the FokI nuclease domain (Figs. S1*C* and 1*C*). Additionally, we set up reactions using native CbAgo or KmAgo to compare their activity to that of the fusion proteins under the same reaction conditions. The native KmAgo showed weaker cleavage activity than FokI-(d)KmAgo on circular and linear targets, while native CbAgo did not show clear target cleavage activity (Figs. S1*C* and 1*C*). These results show that the designed PNFP system enable efficient sequence-specific cleavage of dsDNA target when compared to the PNP system (pAgo-gDNA cleavage of PNA invaded target) reported earlier (43). To prove the compatibility of PNFP system on different targets, we demonstrated the *in vitro* target cleavage activity on pUC19 plasmid bearing the same target region as pMRS plasmid. Results indicating the similar cleavage activity of FokI-(d)CbAgo and FokI-(d)KmAgo on different targets (Fig. S1*D*).

### Optimization of protein and guide DNA concentrations

To determine the optimal concentration of our fusion proteins for efficient target cleavage, we conducted a protein concentration titration assay on γPNA-invaded and noninvaded targets. We determined that 15 nM of FokI-(d)CbAgo or 20 nM of FokI-(d)KmAgo gives results in ideal target cleavage efficiency (Fig. S2*A*). Based on the proof-of-concept experiment, we noticed that FokI-(d)CbAgo and FokI-(d)KmAgo can generate DSBs even with a single guide DNA. Therefore, we conducted another protein concentration titration assay using single guide–loaded FokI-(d)KmAgo using a *Mfe*I-linearized, modified pMRS-6 nt spacer - IN orientation target. Notably, we observed robust cleavage activity with concentrations as low as 5 nM FokI-(d)KmAgo loaded with either guide DNA1 or guide DNA2 (Fig. S2*B*) that presumably binds to only one of the target regions.

Later, we performed a titration experiment using different concentrations of both guide DNAs to determine their optimal concentration. Guide concentrations from 15 nM to 250 nM were ideal for proper cleavage activity with 15 nM FokI-(d)CbAgo or 20 nM FokI-(d)KmAgo (Fig. S3). Interestingly, we observed decreased or no cleavage activity with guide DNA concentrations above 250 nM, indicating that high concentration of guide DNAs could interfere with target cleavage mediated by PNFP systems. We used 50 nM guide DNAs in the rest of experiments to ensure efficient target cleavage activity.

### Role of spacer length and guide DNA orientation on FokI-(d)pAgo cleavage

As observed in previous experiments, reactions using a single guide DNA were sufficient to generate efficient DSBs, and it is difficult to investigate the appropriate guide orientation and proper spacer length *in vitro*. However, we conducted the *in vitro* cleavage experiment to test the cleavage efficiency of FokI-(d)pAgos on *γ*PNA-invaded or noninvaded circular plasmids with different spacer lengths and guide orientations (denoted as “IN,””OUT,” and “SAME”) (Fig. S4*A*). We detected precise DSB generation in all reactions with *γ*PNA-invaded plasmids regardless of guide orientation or spacer length, as we observed bands of the expected size in all cases (Fig. S4*B*). As discussed above, these parameters could be further investigated when applied for the *in vivo* use.

### FokI-(d)pAgo cleavage specificity assays

In initial attempts for PNFP-mediated target cleavage *in vitro*, we noticed nonspecific DNA cleavage and DNA smears, especially in reactions using no guides or nonspecific guides (data not shown). To improve cleavage specificity, we tested different salt concentrations by adding different amounts of NaCl to the rCutSmart buffer. The nonspecific DNA cleavage activity decreased concomitant with the increase of NaCl concentration in all reactions with different guide DNA combinations (Fig. S5), which is consistent with previous studies that reported reduced FokI-mediated nonspecific DNA cleavage at higher salt concentrations (7, 44). When we added 50 mM or 75 mM of additional NaCl to the FokI-(d)CbAgo or FokI-(d)KmAgo reactions, respectively, we observed no cleaved products in guide DNA–free reactions or in reactions with nonspecific guides, while reactions containing a pair of specific guides still presented considerable target cleavage activity (Fig. S5).

**Figure 2:**
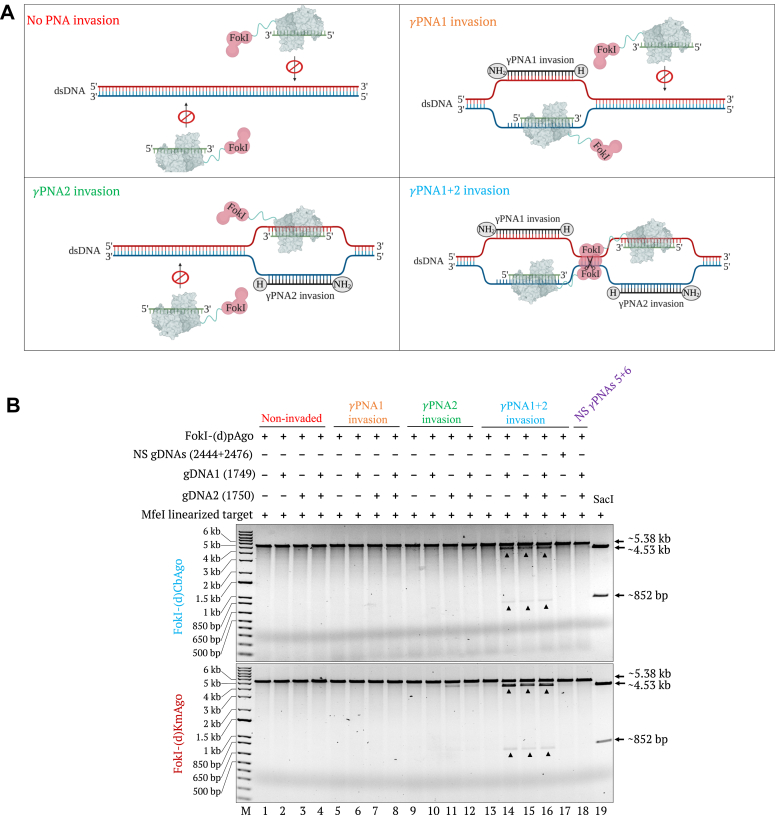
**Effect of PNA invasion on the specificity of FokI-(d)pAgo–mediated cleavage of dsDNA.***A*, schematic representation showing the FokI-(d)pAgo–mediated cleavage of dsDNA noninvaded or invaded with either single *γ*PNA or two *γ*PNAs. When using noninvaded dsDNA target, none of the FokI-(d)pAgo–gDNA complexes can bind to the target regions. When the dsDNA target is invaded with single *γ*PNA, only one of the FokI-(d)pAgo–gDNA complexes can recognize and bind to corresponding target region without forming dimer that is required for target cleavage. When the dsDNA target is invaded with two *γ*PNAs simultaneously at adjacent sites, two FokI-(d)pAgo–gDNA complexes can bind to the unwound ssDNA regions in close proximity, allowing their dimerization and subsequent DSB generation. *B*, gel images showing the FokI-(d)pAgo–mediated cleavage of the *Mfe*I-linearized, modified pMRS-6 nt spacer - IN orientation target either noninvaded (lanes 1–4) or invaded by *γ*PNA1 only (lanes 5–8), *γ*PNA2 only (lanes 9–12), by both *γ*PNA1&2 (lanes 13–16), or by two nonspecific *γ*PNA (lane 18). These targets were then treated with FokI-(d)CbAgo (*top* gel) or FokI-(d)KmAgo (*bottom* gel) preloaded with no guides, a single specific guide, a pair of specific guides, or a pair of nonspecific guides. The cleavage of a target invaded with nonspecific PNAs (*γ*PNA5&6) preloaded with a pair of specific guides by FokI-(d)pAgo was included as a control. The *Sac*I restriction enzyme size control is shown in lane 19. Lane M, 1-kb plus DNA marker.

Next, we tested the cleavage specificity of FokI-(d)pAgo on linear, modified pMRS-6 nt spacer - IN orientation target invaded with *γ*PNA1 only, *γ*PNA2 only, both *γ*PNA1 and *γ*PNA2, two nonspecific *γ*PNAs, or no *γ*PNAs. We treated the targets invaded with the different *γ*PNA combinations as well as the noninvaded target with FokI-(d)pAgos loaded with a single specific guide (guide DNA1 or guide DNA2), a pair of specific guides, or no guides (Fig. 2*A*). We detected strong evidence for DSB generation in reactions from targets invaded with two specific *γ*PNAs (Fig. 2*B*). Meanwhile, we observed very faint bands in the reaction containing *γ*PNA2-invaded target and FokI-(d)KmAgo preloaded with guide DNA2, indicating weak cleavage activity from this PNFP editor on a target invaded with a single *γ*PNA. These results demonstrate that simultaneous invasion of two *γ*PNAs at two adjacent target sites is essential for PNFP editors to generate efficient DSBs at the desired positions, providing an additional layer of specificity for the PNFP editors.

### FokI-(d)pAgo–mediated cleavage of targets with different GC content

Circular plasmids can be cleaved by guide-loaded pAgos at regions with low GC content without any PNA invasions (24, 26, 43). The design of our PNFP editors using dead pAgo proteins fused to the FokI catalytic domain should abrogate this activity. Therefore, we tested FokI-(d)pAgo cleavage activity at regions with low GC content in the noninvaded circular plasmid. FokI-(d)pAgo did not have any cleavage activity at different GC content regions without PNA invasion, whereas FokI-(d)pAgo was catalytically active even at regions with 50% GC content when unwound by adjacent PNA molecules (Fig. S6). This experiment confirmed that FokI-(d)pAgo–mediated target cleavage requires two adjacent PNA molecules and also the specificity of the PNFP editors.

### Time-course assays for *γ*PNA invasion and DNA cleavage

**Figure 3:**
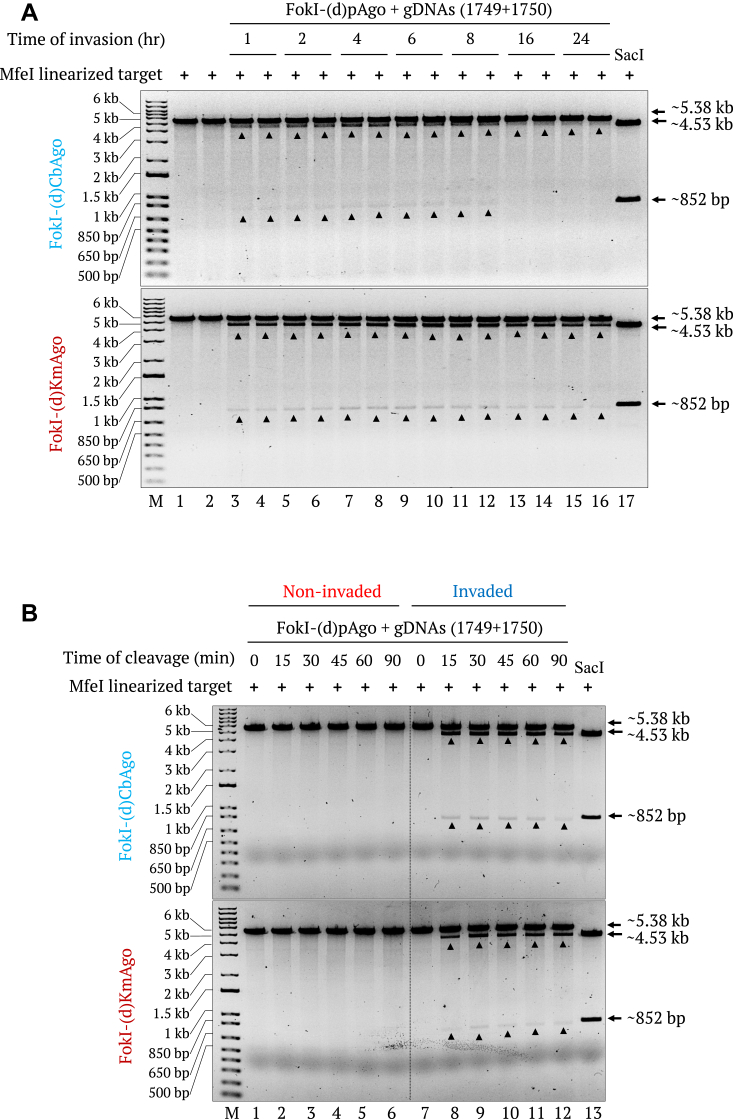
**Time course analysis of γPNA invasion and FokI-(d)pAgo–mediated cleavage of dsDNA.***A*, gel images showing the FokI-(d)CbAgo- (*top*) and FokI-(d)KmAgo- (*bottom*) mediated cleavage of *Mfe*I-linearized, modified pMRS-6 nt spacer - IN orientation target invaded with γPNA1&2 for 1 h, 2 h, 4 h, 6 h, 8 h,16 h, or 24 h (lanes 3–16, two invasion-time reactions were carried out for each time point). The cleavage reaction was conducted for 45 min. Noninvaded targets (lanes 1 and 2) and the *Sac*I restriction enzyme size control (lane 17) were included as controls. *B*, gel images showing the FokI-(d)CbAgo- (*top*) and FokI-(d)KmAgo- (*bottom*) mediated cleavage of a *Mfe*I-linearized noninvaded (lanes 1–6) target and a target invaded with γPNA1&2 for 5 h (lanes 7–12) in cleavage reactions conducted for 0 min, 15 min, 30 min, 45 min, 60 min, or 90 min. The *Sac*I restriction enzyme size control is shown in lane 13. Lane M, 1-kb plus DNA marker.

In previous studies, *γ*PNA invasion into linear DNA observed after variable time points of incubation at 37 °C upon resolvases or pAgo cleavage (43, 45, 46). To determine the optimal reaction time for efficient DSB generation using FokI-(d)pAgos, we performed time course experiments for *γ*PNA invasion and FokI-(d)pAgo–mediated target cleavage. Accordingly, we incubated an *Mfe*I-linearized, modified pMRS-6 nt spacer - IN orientation target with *γ*PNA1 and *γ*PNA2 at 37 °C for 1 h to 24 h before adding FokI-(d)pAgos for an incubation at 37 °C for 45 min. We observed bands indicating site-specific DSB generation starting as early as 1 h following onset of invasion, with peak activity at 4 to 6 h of invasion (Fig. 3*A*). Notably, invasion times longer than 8 h resulted in decreased cleavage efficiency, especially for FokI-(d)CbAgo, suggesting a negative influence from prolonged invasion times. We also conducted a time course cleavage assay using a target invaded with *γ*PNAs for 5 h to determine the ideal time for FokI-(d)pAgo–mediated target cleavage. For both FokI-(d)CbAgo and FokI-(d)KmAgo, we observed robust DSB generation within 15 min when incubating with PNA-invaded targets at 37 °C. Cleavage activity peaked at 30 min of incubation, and prolonged incubation did not appear to improve cleavage performance (Fig. 3*B*).

### FokI-(d)pAgo cleavage temperature assays

**Figure 4:**
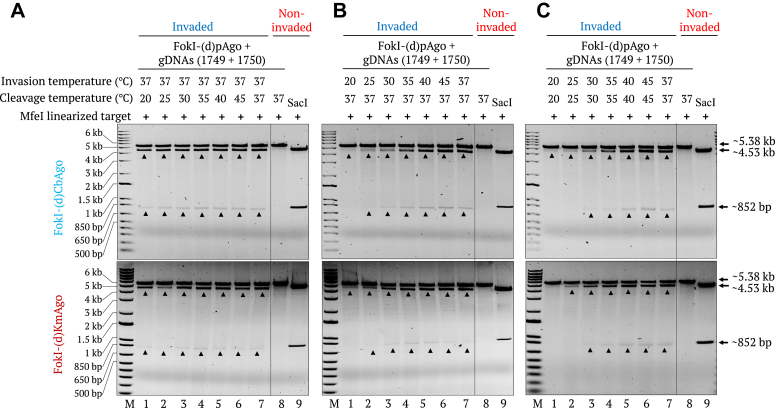
**Effect of different temperatures on γPNA invasion and FokI-(d)pAgo–mediated cleavage of dsDNA.***A*, gel images showing the FokI-(d)pAgo–mediated cleavage of a modified pMRS-6 nt spacer - IN orientation target at different temperatures (20 °C, 25 °C, 30 °C, 35 °C, 40 °C, 45 °C, and 37 °C) after invasion with γPNA1&2 at 37 °C (lanes 1–7). *B*, gel images showing the FokI-(d)pAgo–mediated cleavage of the target at 37 °C after invasion with γPNA1&2 at different temperatures (20 °C, 25 °C, 30 °C, 35 °C, 40 °C, 45 °C, and 37 °C) (lanes 1–7). *C*, gel images showing the FokI-(d)pAgo–mediated cleavage of the target when cleavage and γPNA invasion were conducted at the same temperature (20 °C, 25 °C, 30 °C, 35 °C, 40 °C, 45 °C, and 37 °C) (Lanes 1–7). In all gels, FokI-(d)pAgo–mediated cleavage of noninvaded DNA is shown in lane 8 and the *Sac*I restriction enzyme size control is shown in lane 9. Lane M, 1-kb plus DNA marker.

To assess the potential for PNFP editor-based DNA editing in various organisms that prefer different temperatures, we determined the optimal reaction temperature for *γ*PNA invasion and FokI-(d)pAgo–mediated target cleavage *in vitro*. To this end, we tested the cleavage activity of FokI-(d)CbAgo and FokI-(d)KmAgo on a *γ*PNA1&2-invaded (invasion at 37 °C), linear, modified pMRS-6 nt spacer - IN orientation target at different temperatures ranging from 20 °C to 45 °C. Both fusion proteins presented cleavage activity at all temperatures, with slightly more robust cleavage seen at higher temperatures (Fig. 4*A*). We also tested the optimal temperature for *γ*PNA invasion by incubating *γ*PNA1&2 with the linear target at temperatures ranging from 20 °C to 45 °C, followed by FokI-(d)pAgo–mediated cleavage at 37 °C for 30 min. We observed an apparent increase in cleavage activity with increasing invasion temperature, with the highest cleavage efficiency reached when *γ*PNA invasion was conducted at temperatures above 30 °C (Fig. 4*B*). We then tested the overall performance of the system at different temperatures by conducting *γ*PNA invasion and cleavage at the same temperature, revealing strong cleavage activity at temperatures above 30 °C and lower cleavage efficiency at lower temperatures (Fig. 4*C*). The results exhibited the promise of PNFP systems to be applied in versatile conditions, especially for organisms that require lower temperatures like zebrafish (*Danio rerio*) or *Caenorhabditis elegans.*

### FokI-(d)pAgo activity in different buffers

**Figure 5:**
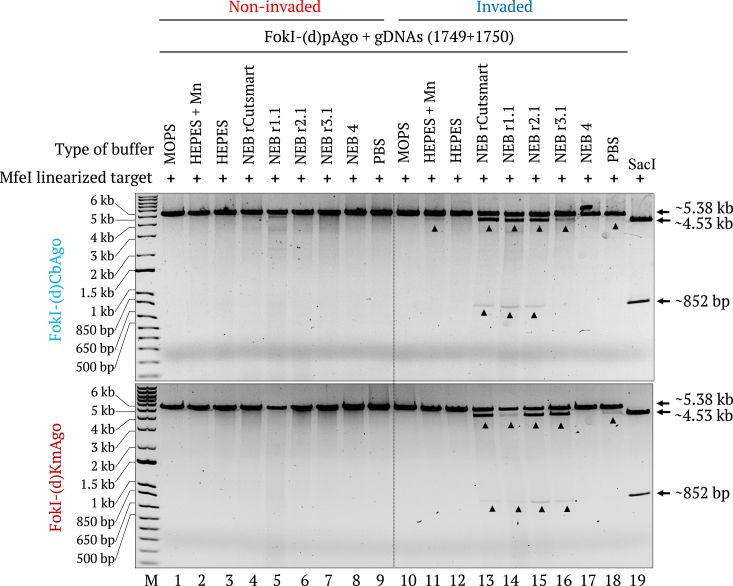
**FokI-(d)pAgo–mediated cleavage in different buffers.** Gel images showing the FokI-(d)CbAgo- (*top*) and FokI-(d)KmAgo- (*bottom*) mediated cleavage of the *Mfe*I-linearized, noninvaded (lanes 1–9) and γPNA1&2-invaded (lanes 10–18) modified pMRS-6 nt spacer - IN orientation target in different buffers. The *Sac*I restriction enzyme size control is shown in lane 19. Lane M, 1-kb plus DNA marker.

In PNFP systems, the appropriate reaction buffer for the FokI-(d)pAgo fusion proteins is essential to obtain effective target cleavage activity. We wished to determine the optimal buffer conditions for the PNFP editors by conducting the *in vitro* cleavage assay in different buffers, including γPNA invasion buffer (MOPS buffer), the cleavage buffer for native pAgos (HEPES + Mn buffer) (43), PBS buffer that simulate *in vivo* conditions, and other related or commonly available buffers, using the MfeI-linearized, modified pMRS-6 nt spacer - IN orientation target. We found that FokI-(d)CbAgo is equally active in NEB rCutSmart, NEB r1.1, and NEB r2.1 buffers, whereas FokI-(d)KmAgo showed robust activity in NEB rCutSmart, NEB r2.1, and NEB r3.1 buffers. Therefore, we used NEB rCutSmart buffer for *in vitro* assays onward (Fig. 5).

### Guide and PNA requirements for efficient DSB generation

**Figure 6:**
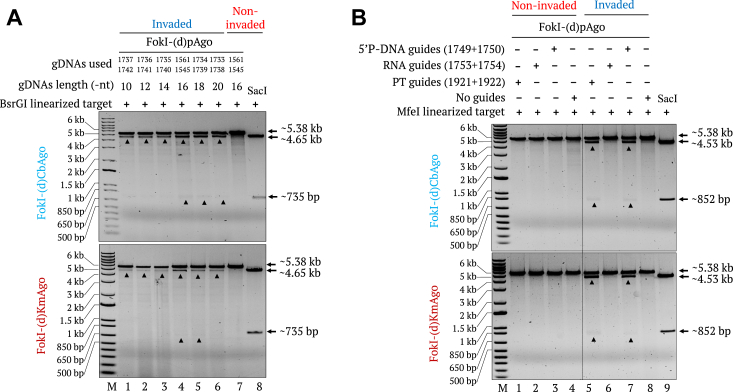
**Effect of guide length and type on FokI-(d)pAgo–mediated cleavage of dsDNA.***A*, gel images showing the effect of guide length on FokI-(d)CbAgo- (*top*) and FokI-(d)KmAgo- (*bottom*) mediated cleavage of a *Bsr*GI-linearized, modified pMRS-γPNA3/4-6 nt spacer target. The target invaded with γPNA3&4 was incubated with FokI-(d)pAgo preloaded with a pair of guide DNAs of 10 nt, 12 nt, 14 nt, 16 nt, 18 nt, or 20 nt in length (lanes 1–6). Noninvaded target incubated with FokI-(d)pAgo preloaded with 16-nt-long guides was included as a control (Lane 7). The *Sac*I restriction enzyme size control is shown in lane 8. Lane M, 1-kb plus DNA marker. *B*, gel images showing the FokI-(d)CbAgo- (*top*) and FokI-(d)KmAgo- (*bottom*) mediated cleavage of the *Mfe*I-linearized, modified pMRS-6 nt spacer - IN orientation target employing different types of guides. The noninvaded (lanes 1–4) or γPNA1&2-invaded (lanes 5–8) target was incubated with FokI-(d)pAgo preloaded with 5′-phosphorylated phosphorothioated guides (PT-guides; PT bond at every position) (lanes 1 and 5), 5′-phosphorylated RNA guides (RNA guides) (lanes 2 and 6), 5′-phosphorylated DNA guides (5′ P-DNA guides) (lanes 3 and 7), or no guides (lanes 4 and 8). The *Sac*I restriction enzyme size control is shown in lane 9. Lane M, 1-kb plus DNA marker.

We investigated the requirements of the guides and PNAs for efficient target cleavage activity by FokI-(d)CbAgo and FokI-(d)KmAgo. To establish the best-suited guide length, we loaded each fusion protein with different guide DNAs ranging from 10 nt to 20 nt and examined their activity on linear targets invaded with a pair of 20-nt-long *γ*PNAs. We observed greater cleavage efficiency when using the longer guide DNAs (16–20 nt), with the best activity obtained when using 16-nt-long guides (Fig. 6*A*).

Many studies have reported the use of pAgos mainly with short ssDNA guides with 5′ end phosphorylation, while RNA guides tend to result in weak activity (47). To demonstrate the versatility of guide design, we loaded the FokI-(d)pAgos with different types of guides, including 5′-phosphorylated phosphorothioated guides (PT-guides), 5′-phosphorylated RNA guides (RNA guides), and 5′-phosphorylated DNA guides (5′ P-DNA guides). Cleavage results showed that FokI-(d)pAgos loaded with 5′ P-DNA guides and PT-guides efficiently generate DSBs, while the RNA guides did not show any cleavage activity (Fig. 6*B*).

**Figure 7:**
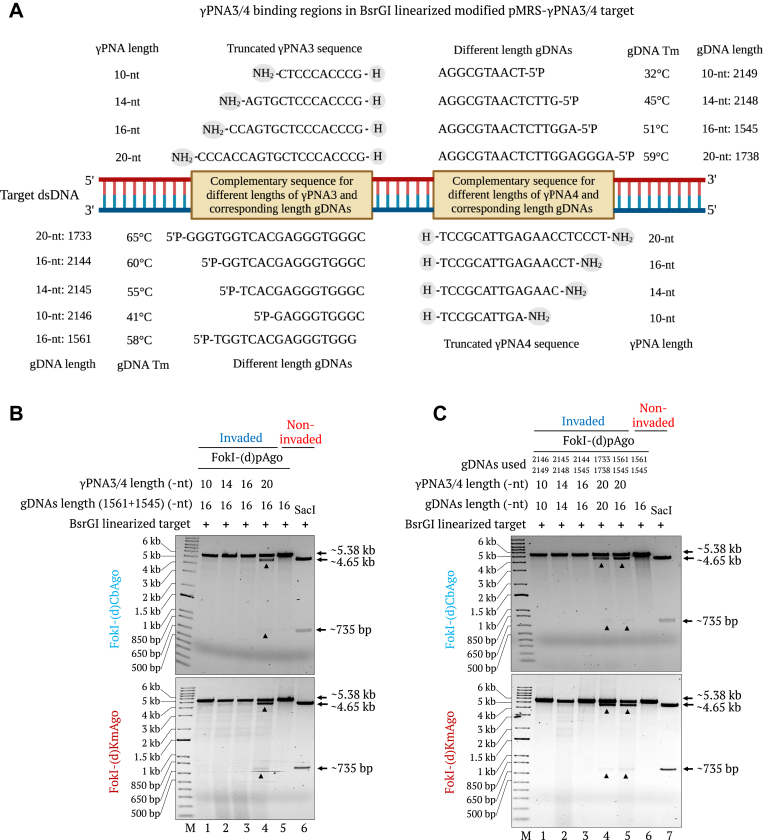
**Effect of PNA and guide length on FokI-(d)pAgo–mediated cleavage of dsDNA.***A*, diagrams showing the target region of modified pMRS-γPNA3/4-6 nt spacer target and invasion sites of *γ*PNA3, *γ*PNA4, truncated versions of *γ*PNA3&4, and corresponding guide DNAs. Tm indicates melting temperature. *B*, gel images showing the FokI-(d)CbAgo- (*top*) or FokI-(d)KmAgo- (*bottom*) mediated cleavage of the *Bsr*GI-linearized, modified pMRS, γPNA3/4-invaded, 6-nt spacer, IN orientation target invaded by 10-nt (lane 1), 14-nt (lane 2), 16-nt (lane 3), or 20-nt (lane 4) -long *γ*PNA3 and *γ*PNA4. FokI-(d)pAgo proteins were preloaded with 16-nt-long guide DNAs (1561 and 1545). Noninvaded target cleavage with FokI-(d)pAgos (lane 5) and *Sac*I-digested (lane 6) samples were included as controls. Lane M, 1-kb plus DNA marker. *C*, gel images showing the FokI-(d)CbAgo- (*top*) or FokI-(d)KmAgo- (*bottom*) mediated cleavage of the target invaded by 10-nt (lane 1), 14-nt (lane 2), 16-nt (lane 3), or 20-nt (lane 4) -long *γ*PNA3 and *γ*PNA4 with different guide DNA lengths. FokI-(d)pAgo proteins were pre-loaded with 10-nt, 14-nt, 16-nt, or 20-nt-long guide DNAs. Noninvaded target cleavage with FokI-(d)pAgos (lane 5) and *Sac*I-digested (lane 6) samples were included as controls. Lane M, 1-kb plus DNA marker.

We also evaluated the optimal length of the *γ*PNAs required for PNFP editors. We tested 10-nt, 14-nt, 16-nt, and 20-nt-long *γ*PNAs for target invasion with FokI-(d)pAgo proteins preloaded with guide DNAs of differing lengths (10-nt, 14-nt, 16-nt, and 20-nt) (Fig. 7*A*). We detected cleavage of targets invaded with different truncated PNAs using FokI-(d)pAgo preloaded with 16-nt-long guide DNAs only when the *γ*PNAs were 20-nt long (Fig. 7*B*). Similarly, when we used *γ*PNAs and guide DNAs of the same lengths, we observed cleavage only with the 20-nt-long *γ*PNAs (Fig. 7*C*). Therefore, 20-nt *γ*PNAs are needed for proper PNFP editor function, as reactions with shorter *γ*PNAs presented no clear band of the expected size, regardless of guide length.

### Effect of guide DNA mismatches on PNFP editors

The guide molecule for Ago proteins can be subdivided into different segments, usually including the 5′ nucleotide (anchor) that is attached to the MID domain-binding pocket, the seed region (positions 2–8) crucial for target recognition, followed by the central part surrounding the pAgo cleavage site, the 3′ supplementary region, and the 3′ tail (from position 16 onward) (23, 25, 48). The 3′ end of the guide is fixed in the pocket of the PAZ domain (23). Mismatches in different regions of the guide molecule have various effects on the target recognition and cleavage activity of eukaryotic Agos and pAgos (24, 26, 49). To investigate the effect of guide mismatches on (d)pAgo-mediated target recognition, we designed guide DNAs bearing 1 to 6 nt mismatches at each region; we used pairs of guide DNAs with corresponding mismatches for assays (Table 1).

**Table 1:** Sequences of full complementary and mismatched guide DNA 1 and guide DNA 2

Number of mismatched nucleotides	Guide DNA name	Mismatch at guide anchor region	Mismatch at guide seed region	Mismatch at guide central region	Mismatch at guide supplementary region	Mismatch at guide tail region
Full complementary	Guide DNA 1	5′TCCGAGCAGAAGAAGA3′				
	Guide DNA 2	3′CGAGGGTAGTGTAGTT5′				
1-nt mismatch	Mismatch guide 1	5′ACCGAGCAGAAGAAGA3′	5′TCCGAGCAGAAGAAGA3′	5′TCCGAGCACAAGAAGA3′	5′TCCGAGCAGAAGTAGA3′	5′TCCGAGCAGAAGAAGT3′
	Mismatch guide 2	3′CGAGGGTAGTGTAGTA5′	3′CGAGGGTAGTGTTGTT5′	3′CGAGGGTTGTGTAGTT5′	3′CGACGGTAGTGTAGTT5′	3′GGAGGGTAGTGTAGTT5′
2-nt mismatch	Mismatch guide 1	5′AGCGAGCAGAAGAAGA3′	5′TCCGTGCAGAAGAAGA3′	5′TCCGAGCTCAAGAAGA3′	5′TCCGAGCAGAACTAGA3′	5′TCCGAGCAGAAGAACT3′
	Mismatch guide 2	3′CGAGGGTAGTGTAGAA5′	3′CGAGGGTAGTGATGTT5′	3′CGAGGGTTCTGTAGTT5′	3′CGACCGTAGTGTAGTT5′	3′GCAGGGTAGTGTAGTT5′
3-nt mismatch	Mismatch guide 1	5′AGGGAGCAGAAGAAGA3′	5′TCCGTCCAGAAGAAGA3′	5′TCCGAGCTCTAGAAGA3′	5′TCCGAGCAGATCTAGA3′	5′TCCGAGCAGAAGATCT3′
	Mismatch guide 2	3′CGAGGGTAGTGTACAA5′	3′CGAGGGTAGTCATGTT5′	3′CGAGGGATCTGTAGTT5′	3′CGACCCTAGTGTAGTT5′	3′GCTGGGTAGTGTAGTT5′
4-nt mismatch	Mismatch guide 1	5′AGGCAGCAGAAGAAGA3′	5′TCCGTCGAGAAGAAGA3′	5′TCCGAGGTCTAGAAGA3′	5′TCCGAGCAGTTCTAGA3′	5′TCCGAGCAGAAGTTCT3′
	Mismatch guide 2	3′CGAGGGTAGTGTTCAA5′	3′CGAGGGTAGACATGTT5′	3′CGAGGGATCAGTAGTT5′	3′CGACCCAAGTGTAGTT5′	3′GCTCGGTAGTGTAGTT5′
5-nt mismatch	Mismatch guide 1	5′AGGCTGCAGAAGAAGA3′	5′TCCGTCGTGAAGAAGA3′	5′TCCGAGGTCTTGAAGA3′	5′TCCGAGCACTTCTAGA3′	5′TCCGAGCAGAACTTCT3′
	Mismatch guide 2	3′CGAGGGTAGTGATCAA5′	3′CGAGGGTACACATGTT5′	3′CGAGGCATCAGTAGTT5′	3′CGACCCATGTGTAGTT5′	3′GCTCCGTAGTGTAGTT5′
6-nt mismatch	Mismatch guide 1	5′AGGCTCCAGAAGAAGA3′	5′TCCGTCGTCAAGAAGA3′	5′TCCGACGTCTTGAAGA3′	5′TCCGAGCTCTTCTAGA3′	5′TCCGAGCAGATCTTCT3′
	Mismatch guide 2	3′CGAGGGTAGTCATCAA5′	3′CGAGGGTTCACATGTT5′	3′CGAGGCATCACTAGTT5′	3′CGACCCATCTGTAGTT5′	3′GCTCCCTAGTGTAGTT5′
Full mismatch	Nonspecific guide 1	5′AGGCTCGTCTTCTTCT3′				
	Nonspecific guide 2	3′GCTCCCATCACATCAA5′				

**Figure 8:**
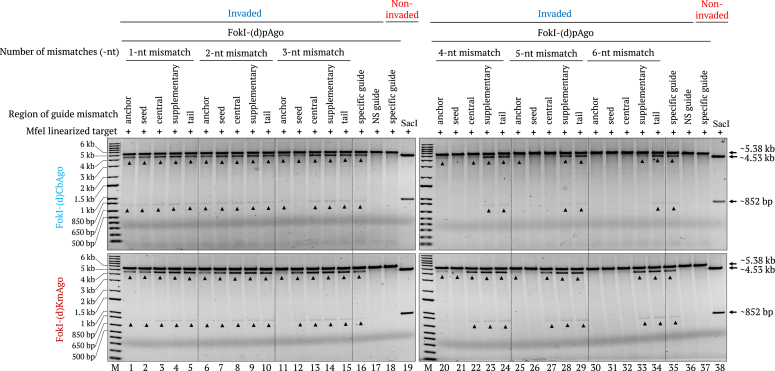
**Effect of guide mismatches on FokI-(d)pAgo–mediated cleavage of dsDNA.** Gel images showing the FokI-(d)CbAgo- (*top*) or FokI-(d)KmAgo- (*bottom*) mediated cleavage of the γPNA1&2-invaded, modified pMRS-6 nt spacer - IN orientation target. FokI-(d)pAgo proteins were preloaded with different gDNAs containing 1 to 6 mismatches at different positions in the guide architecture. FokI-(d)pAgo–mediated cleavage on the γPNA1&2-invaded target using specific guides (lanes 16 and 35) and nonspecific guides (lanes 17 and 36), and on a noninvaded target using specific guides (lanes 18 and 37), together with the *Sac*I-digested (noninvaded) samples (lanes 19 and 38) were included as controls. Lane M, 1-kb plus DNA marker.

When using FokI-(d)CbAgo or FokI-(d)KmAgo, one or two mismatches within any region did not clearly influence cleavage performance. With FokI-(d)CbAgo, we observed significantly decreased activity when introducing three or more mismatches into the seed region of the guide DNAs. Four mismatches in the anchor and central regions and six mismatches in the supplementary region largely compromised cleavage activity, while mismatches until 6-nt in the tail region were well tolerated (Fig. 8). These results indicate that positions 4′ to 8′ in the seed region of the guide DNAs are critical for FokI-(d)CbAgo–mediated target cleavage. For FokI-(d)KmAgo, the target cleavage activity was largely diminished when using guide DNAs with more than two or three mismatches in the anchor or seed region, respectively, indicating that the anchor and seed regions of the guide DNAs are essential for target recognition by (d)KmAgo. By contrast, six mismatches in the supplementary or tail regions of the guide DNA were well tolerated and even slightly stimulated cleavage activity (Fig. 8). To conclude, FokI-(d)KmAgo showed lower tolerance to mismatches in the first seven nucleotide positions from the 5′ end of the guide DNA, covering most of the seed region, while the specificity of the remaining nine positions did not appear to substantially influence overall activity.

### Cleavage site determination of PNFP editors

**Figure 9:**
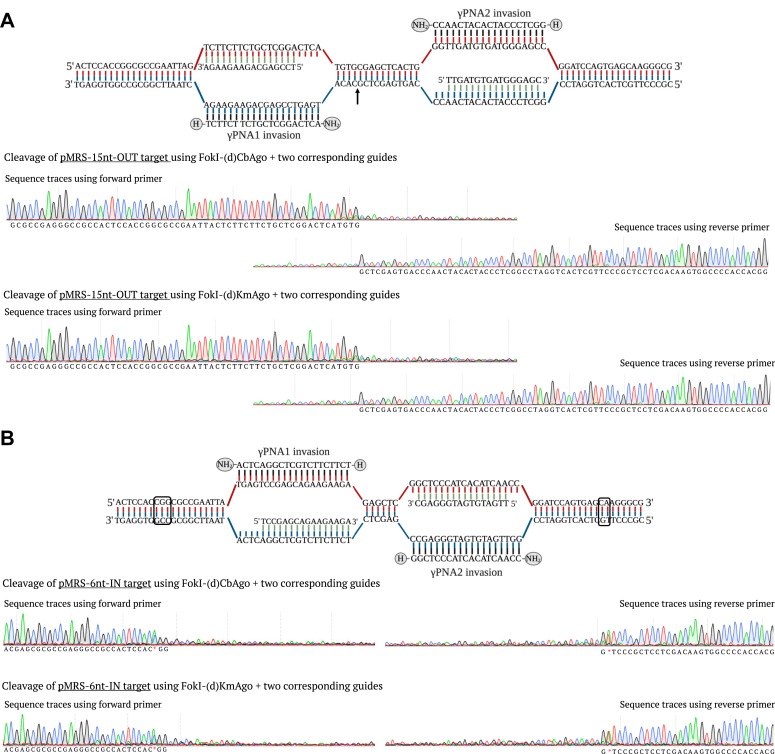
**Cleavage site identification of FokI-(d)pAgo on different targets.***A*, *Bsa*I-linearized γPNA1&2-invaded, modified pMRS-15 nt spacer - OUT orientation target bound with guide DNA1 and guide DNA2 represented on *top*. Sanger sequencing reads of FokI-(d)CbAgo and FokI-(d)KmAgo cleaved products using forward primer and reverse primer represented below. *B*, *Bsa*I-linearized, γPNA1&2-invaded, modified pMRS-6 nt spacer - IN orientation target bound with guide DNA1 and guide DNA2 represented on *top*. Sanger sequencing reads of FokI-(d)CbAgo and FokI-(d)KmAgo cleaved products using forward primer and reverse primer represented below. *Arrows* indicate the approximate cleavage positions of FokI-(d)pAgo. *Arrow marks* and *box* indicate the approximate cleavage positions of FokI-(d)CbAgo and FokI-(d)KmAgo; *asterisk* is the mismatched nucleotide read after Sanger sequencing.

To detect the cleavage position of the PNFP system, we conducted the cleavage site determination assay by Sanger sequencing of the PNFP cleaved products. First, we performed the *in vitro* cleavage of different *Bsa*I-linearized targets using FokI-(d)CbAgo or FokI-(d)KmAgo complexed with single or two guide DNAs, independently (Fig. S7, *A*–*C*). Later, the two released fragments were subjected to Sanger sequencing. When using two guides on the pMRS-γPNA1/2-15 nt spacer OUT target (15-OUT target), we observed cleavage in between the two *γ*PNA-binding regions, at around 4 bp downstream of the last nucleotide of *γ*PNA1 invasion region (Fig. 9*A*). In contrast to 15-OUT target, when using the pMRS-γPNA1/2-6 nt spacer IN target (6-IN target), the sequencing reads showed two sites outside of the *γ*PNA-binding regions, with one site being observed at around 11 to 13 bp upstream of the first nucleotide of *γ*PNA1-binding region and the other one at about 13 to 14 bp downstream of the last nucleotide of *γ*PNA2-binding region (Fig. 9*B*). For both 15-OUT and 6-IN targets, the cleavage sites were identified close to the two *γ*PNA invasion regions. We also determined the cleavage positions using single guide DNA (either gDNA1 or gDNA2 only) on the 6-IN target, and we observed cleavage outside the two PNA invasion regions, similar to the findings in the two guide DNA–mediated cleavage reactions (Fig. S8, *A* and *B*). Consistent with our findings in the proof-of-concept session, FokI-(d)pAgo coupled with single guide DNA can generate DSB *in vitro*, whereas higher cleavage specificity could be obtained when using the two guide DNA molecules simultaneously.

Later, we examined the effect of FokI-(d)pAgo on ssDNA target by incubating a 95-nt long ssDNA bearing a guide DNA 4 targeting region with corresponding guide complexed FokI-(d)CbAgo or FokI-(d)KmAgo. Cleavage reactions using native CbAgo or KmAgo on the same ssDNA target were also conducted as positive controls, generating two bands (52-nt and 43-nt) in case of site-specific cleavage (Fig. S9*A*). When using different concentrations of FokI-(d)CbAgo or FokI-(d)KmAgo, no sign of site-specific target cleavage was observed, compared with positive controls (Fig. S9*B*). Instead, nonspecific DNA degradation was observed when increasing the concentration of FokI-(d)KmAgo, which is also observed when incubating the ssDNA target with FokI nuclease domain (Fig. S9, *B* and *C*), indicating that dsDNA is required for target cleavage mediated by FokI-(d)pAgo.

## Discussion

Directed by short DNA or RNA guides, pAgos enable target-specific DNA cleavage in a PAM-independent manner, showing higher flexibility than CRISPR-based systems (50). Recently, we developed PNP editors harnessing PNAs that invade dsDNAs with high specificity to facilitate unwinding the target region for pAgo-mediated DNA cleavage (43). This work achieved site-specific target cleavage at physiological temperature regardless of GC content and DNA form *in vitro*, meeting the promise of the PNP editors for use in genome editing with high specificity and versatility. However, guide-independent DNA cleavage activity of pAgos might be a challenge for the deployment of PNP editors *in vivo*. In the current work, we improved on this system by using catalytically dead pAgos for sequence-specific target recognition and FokI as a robust nuclease. In the resulting PNFP system, a pair of guide-loaded fusion proteins simultaneously bind to two DNA segments exposed by *γ*PNA invasion, in sufficiently close proximity to allow the dimerization of the FokI subdomains, followed by FokI cleavage of the target site to generate DSBs. We expect that the requirement for forming a functional dimer improves the specificity of PNFP editors. In addition, use of the FokI nuclease domain may have improved cleavage efficiency compared to previous designs using native pAgos. Moreover, the risk of nonspecific chopping activity can be minimized by using (d)pAgos lacking cleavage function.

The predicted improvements in the design resulted in improved cleavage efficiency and specificity *in vitro*. For example, we confirmed the sequence-specific cleavage of the PNFP system on circular and linearized plasmids. The system could also generate considerable DSBs only when the target is invaded with two *γ*PNAs simultaneously. Meanwhile, no evidence of FokI-(d)pAgo–mediated cleavage was detected on circular plasmids without *γ*PNA invasion even when targeting regions with low GC content. Besides, 20-nt *γ*PNAs invaded targets efficiently and the best activity was obtained using fusion proteins loaded with 16-nt guide DNAs. Moreover, the PNFP editors were able to use 5′ P-DNA guides and 5′ PT-guides, enabling efficient DSB generation from 30 °C to 45 °C. In addition, the time-course results confirmed that efficient target cleavage could be obtained within 15 min of incubation at 37 °C, with a peak at 30 min. Under the same conditions, FokI-(d)pAgos showed significantly higher cleavage efficiency than intact fully active pAgos. The cleavage site identification results of both pMRS-γPNA1/2-15 nt spacer OUT and pMRS-γPNA1/2-6 nt spacer IN plasmids confirmed that the PNFP systems produced cleavage within and outside of the two γPNA invasion regions, respectively. FokI-(d)CbAgo or FokI-(d)KmAgo produced a cut in between the two γPNA invasion regions on the 15-OUT target and multiple cuts outside of the two γPNA invasion regions on the 6-IN target. The reason for the different cleavage positions on these two different plasmids needs to be further investigated.

Theoretically, the proper orientation and the appropriate spacer length between the two target sites are critical to ensure FokI domain dimerization. However, we obtained robust target cleavage when using fusion proteins loaded with a single guide, presumably binding to only one of the target regions. A similar phenomenon was observed during the *in vitro* establishment of TALENs, where DNA carrying a single binding site was effectively cleaved by the corresponding TALEN monomers (8). Several hypotheses can explain cleavage with a single guide: (1) the DNA-bound fusion protein can recruit a second freely available FokI-(d)pAgo from the reaction mix *in vitro*; (2) the fusion protein bound to its recognition site can dimerize with another monomer bound nonspecifically to the same DNA molecule; (3) two fusion proteins bound to two DNA molecules can dimerize to generate DSBs. For the first two possibilities, the formed dimers are likely to be very unstable, and relatively high concentrations of fusion proteins should be required to generate effective target cleavage (7). Considering that very low concentration of FokI-(d)pAgos loaded with a single guide resulted in efficient DSB generation, the first two hypotheses are likely not the main mechanisms for single guide–mediated target cleavage. Given these reasons, the third model may best explain the effective target cleavage activity observed when using a single guide, as also suggested in the TALEN work (8). A similar model was also proposed for DNA cleavage mediated by intact FokI *in vitro*, where a FokI monomer bound to its recognition site recruits another FokI monomer bound to a second DNA molecule, generating a DSB in the first DNA molecule (51, 52). Despite the robust activity shown *in vitro*, this phenomenon is unlikely to happen *in vivo* because of much lower concentrations of fusion proteins and targets, as also demonstrated in the TALEN work with single-strand annealing assays in yeast (*Saccharomyces cerevisiae*) that a single binding site was insufficient for TALEN monomers to generate target cleavage effectively (8).

The specificity of the PNFP editors depends on the specificity of both *γ*PNA invasion and guide DNA–directed DNA recognition. In this study, we tested guide specificity by introducing mismatches at different regions along their sequence. We established that cleavage activity was largely impaired when introducing mismatches into the seed region of the guide DNAs, especially at positions 4 to 8 from the 5′ end for FokI-(d)CbAgo and positions 1 to 7 from the 5′ end for FokI-(d)KmAgo, while mismatches in regions close to the 3′ end were largely tolerated. This finding is not consistent to our previous observation that intact CbAgo and KmAgo are highly sensitive to mismatches in the central and supplementary regions (43). As reported for many Argonaute proteins, the seed region plays an important role in target recognition, with high sensitivity to even single nucleotide mismatches that markedly decrease target binding (24, 53, 54, 55). In addition, mismatches near the cleavage site (central region) usually abolish cleavage activity, as shown in our previous work as well (43, 56, 57, 58). Since the (d)pAgos only function in target recognition without any cleavage activity, it is not surprising to observe a significantly lower tolerance to mismatches in the seed region rather than in the central region. However, why mutations in the PIWI domain is responsible for this change remain to be explored.

In conclusion, we developed a PNFP editing system for precise dsDNA editing. The FokI-(d)pAgo proteins presented robust target-specific cleavage activity under mesophilic conditions independently of the DNA form of their target *in vitro*. We predict several potential advantages for these PNFP editors over the previous PNP design when applied *in vivo*: (1) PNFP editors may limit the risk caused by the guide-independent DNA cleavage activity of intact pAgos; (2) PNFP editors enhance editing efficiency in an environment with relatively low concentrations of both protein and target; (3) PNFP editors will improve targeting specificity and minimize off-target activity derived from the requirement of dimer formation. Unlike many CRISPR-based platforms, the PNFP editors allow PAM-independent sequence recognition that could be easily reprogrammed for various targets with fewer constraints. Additionally, designs based on smaller pAgos than Cas proteins may provide higher efficiency in packaging and delivery.

Despite the above potential benefits, several challenges from this design remain when applied *in vivo*. The efficiency of *γ*PNA invasion is compromised under physiological conditions with high salt concentrations and ionic strength (59), and *γ*PNA invasion in cellular environments may be challenging. Using *γ*-modified tail clamp PNA (*γ*tcPNA) or *γ*PNA with miniature poly(ethylene glycol) (miniPEG) modification in combination with nanoparticle-based methods like polylactic-co-glycolic acid was previously suggested to improve dsDNA invasion. In addition to PNAs, other elements or accessory proteins like helicases and ssDNA-binding protein proteins should be considered for assisting with pAgo-DNA binding (24, 30, 60). Another problem is that the FokI-(d)pAgo proteins may acquire naturally occurring short nucleotides like microRNAs or degraded DNA fragments as nonspecific guides. This problem may be overcome by delivering guide preloaded fusion proteins into cells directly, although the method for efficient delivery remains to be established.

The establishment of PNFP editors expands the promise of pAgos to new genome-editing platforms. As a superfamily, pAgo proteins offer a repertoire of candidates with different preferences on guide and target that could be employed with high flexibility in various species. Besides programmable nucleases, we hope that this work will also broaden the deployment of pAgos for different biotechnological applications *in vivo*. For example, catalytically dead pAgos that allow target recognition may be fused to other components to support site-specific base editing, regulation of gene expression, epigenetic editing, prime-editing, *in vivo* imaging, to name a few. We envision that pAgo-based platforms will serve as powerful alternatives to CRISPR/Cas systems in different fields.

## Experimental procedures

### Construction of FokI-(d)pAgo expression vectors and protein purification

#### FokI-(d)CbAgo

The FokI-(d)CbAgo expression vector was designed *in silico* by subcloning the codon-optimized sequence encoding an NLS fused to FokI, the flexible linker, and the catalytically inactive (d)CbAgo into the His6-TwinStrep-SUMO expression vector; the codon-optimized sequence was ordered from GenScript (File S1*A*). All protein sequences are listed in Table S1.

For purification of recombinant FokI-(d)CbAgo protein, the FokI-(d)CbAgo expression vector was transformed into *Escherichia coli* strain BL21; positive colonies were cultured in LB medium containing 100 μg/ml ampicillin for 12 h at 37 °C with shaking at 180 rpm. Then, 20 ml of the above starter culture was used to inoculate 1 L of Terrific Broth medium (IBI Scientific) containing 100 μg/ml ampicillin. Then, the culture was placed in four flasks (4 × 1 L of culture in each 6-L flask) for protein purification. The inoculated cultures were incubated at 37 °C until A_600_ reached ∼0.5. Then, the cultures were placed at 4 °C for 30 min and protein expression was induced by adding IPTG to a final concentration of 0.5 mM. The cultures were incubated for 18 h at 20 °C with shaking at 180 rpm. Cell pellets were harvested by centrifugation at 4 °C for 20 min at 4000 rpm (Thermo Fisher Scientific, Sorvall LYNX 6000). Then, cell pellets were resuspended and lysed in four volumes of lysis buffer (50 mM Tris–HCl pH 7.5, 300 mM NaCl, 4.5 mM MgCl_2_, 5% [v/v] glycerol, 20 mM imidazole, 1 mg/ml lysozyme [L6876, Sigma], EDTA-free protease inhibitor (1 tablet per 50 ml lysis buffer) [Thermo Fisher Scientific, A32953], 0.5 mM PMSF, 1 mM TCEP [tris(2-carboxyethyl)phosphine], and Benzonase Nuclease (1 μl for 100 ml lysis buffer) (Merck, E1014-5KU)) for 45 min at 4 °C.

The cell lysate was sonicated (Qsonica Q700) to release proteins before centrifugation of the mixture at 18,000 rpm and 4 °C for 45 min (Thermo Fisher Scientific, Sorvall LYNX 6000). The supernatant was filtered through a 0.45 μM bottletop vaccum filter (Thermo Fisher Scientific – 2914545) and applied onto a Ni-NTA column (HisTrap HP, 5 ml GE Healthcare) *via* affinity chromatography using ÄKTA Pure (GE Healthcare). The unbound proteins were removed by washing the column with buffer A (50 mM Tris–HCl pH 7.5, 500 mM NaCl, 5% [v/v] glycerol, 20 mM imidazole, 1 mM TCEP). Recombinant 6xHis-SUMO-NLS-FokI-(d)CbAgo was eluted using buffer B (50 mM Tris–HCl pH 7.5, 500 mM NaCl, 300 mM imidazole, and 1 mM TCEP). The eluted protein was collected and treated with SUMO protease to remove the 6xHis-SUMO tag during overnight dialysis at 4 °C in dialysis buffer (50 mM Tris–HCl pH 7.5, 100 mM NaCl, 5% [v/v] glycerol, 1 mM TCEP). The resulting solution was loaded onto an Ni-NTA column and treated with the same buffer A and buffer B mentioned above. The tag-free protein was collected and exchanged with low-salt buffer (50 mM Tris–HCl pH 7.5, 100 mM NaCl, 1 mM TCEP) using Amicon Ultra-15 Centrifugal Filter Units (50 kDa NMWL, UFC905024, Millipore) and then applied onto a cation exchange column (HiTrap SP HP, 5 ml GE Healthcare), washed with low-salt buffer, and eluted in the same buffer with a linear NaCl gradient (0.1–2.0 M). The protein was collected and further purified by size-exclusion chromatography on an S200 column (GE Healthcare) in gel filtration buffer (25 mM Tris–HCl pH 7.5, 100 mM NaCl, 0.1 mM EDTA, 1 mM TCEP, and 10% [v/v] glycerol). After examination by SDS-PAGE electrophoresis, the fractions containing the protein were pooled, concentrated to ∼0.2 mg/ml, snap-frozen, and stored at −80 °C.

#### FokI-(d)KmAgo

The protein expression vector was constructed using the pET28a-6Xhis-HRV3C-KmAgo backbone (gift from prof. Lixin Ma at Hubei University) with the coding sequence of *KmAgo* from *K. massiliensis* replaced with that encoding NLS-FokI-(d)KmAgo (ordered from GenScript) (File S1*B*). The protein was purified as previously reported by Liu Y *et al.* (26). All protein sequences are listed in Table S1.

### Design and synthesis of *γ*PNAs

All *γ*PNAs used in this study have been previously reported (37, 61) and were custom synthesized by PANAGENE Inc. based on general PNA synthesis guidelines (https://www.pnabio.com/support/PNA_Tool.htm). All *γ*PNAs were *γ*-modified with alanine molecules at all bases, and three lysine moieties were added to facilitate solubility (Table S2).

### Cloning of *γ*PNA invading target regions into pMRS plasmids

All target sites were designed as forward and reverse oligonucleotides with overhangs corresponding to *Bam*HI and *Eco*RI restriction sites that were purchased from Integrated DNA Technologies Inc, listed in Table S3. The forward and reverse oligonucleotides of each target site were phosphorylated using T4 PNK (Promega) and annealed to form dsDNA fragments. The pMRS plasmid was digested with *Bam*HI-HF and *Eco*RI-HF in 1× rCutSmart buffer (NEB) for 8 h and purified using a QIAquick PCR Purification Kit (28104). The phosphorylated annealed fragments above were ligated with the purified backbone usingT4 DNA Ligase (Promega) in 1× T4 DNA Ligase Reaction Buffer (Promega; catalog #M1801) for 3 h at 23 °C, followed by 2 h at 16 °C, and kept at 4 °C before use. For each target plasmid, 2 μl of the ligation reaction was transformed into *E. coli* competent cells (Thermo Fisher Scientific, One Shot TOP10 Chemically Competent *E. coli*, C404010) using a heat-shock method; the transformed cells were plated on agar plates containing 50 μg/ml kanamycin. The plasmids were isolated from bacterial liquid cultures using a QIAprep Spin Miniprep Kit (Qiagen, 27106) and confirmed by Sanger sequencing using the primers listed in Table S4.

### *γ*PNA invasion and FokI-(d) pAgo-mediated *in vitro* cleavage assay

Both circular targets and linear targets were used for the *in vitro* validation of PNFP editors. The linear plasmid DNA targets were generated by restriction digestion using *Mfe*I and purified with a QIAquick PCR Purification Kit (28104) as per the manufacturer’s guidelines. Prior to the cleavage assay, *γ*PNA invasion was conducted by incubating 200 ng of DNA targets with 100 nM of each *γ*PNA (*γ*PNA1 and *γ*PNA2) in 1× MOPS buffer (20 mM MOPS pH 7.0, 5 mM CH₃COONa, 1 mM EDTA) in a 10-μL reaction volume at 37 °C for 1 h or 5 h for circular or linear plasmids, respectively. Keeping the reaction components consistent, the type of PNA (different lengths), invasion time points, and temperatures were modified for the corresponding experiments. Detailed invasion protocol is provided in Supplementary methods section.

The FokI-(d)pAgos–mediated cleavage was conducted in two steps: (1) assembly of FokI-(d)pAgo with the corresponding guide DNA and (2) incubating a pair of guide-loaded FokI-(d)pAgos with target DNA. First, the FokI-(d)pAgo was loaded with two guide DNAs independently in two half reactions in 1 × NEB rCutSmart buffer (NEB; catalog #B6004S) at 37 °C for 15 min. Then, 8 μl of each half reaction (15 nM of FokI-(d)CbAgo, 20 nM of FokI-(d)KmAgo, and 50 nM of guide DNA final unless otherwise mentioned) was combined and incubated with 4 μl of PNA-invaded target (80 ng target DNA final) in a 20-μL reaction volume at 37 °C for 30 min. For the circular target, 1 μl of the appropriate restriction enzyme was added into the reaction and incubated at 37 °C for 30 min. Next, to remove FokI-(d)pAgo, 1 μl of proteinase K (Invitrogen; catalog # 25530049) was added to each reaction and incubated at 37 °C for 30 min. After incubation, the reactions were mixed with 4 μl of 6 × Gel Loading Dye Purple (NEB; catalog #: B7024S) and loaded onto a 0.9% (w/v) agarose gel with GelRed and electrophoresed for 1 h at 145 V for 30 min. Finally, the gel was visualized using a FluorChemQ Gel Doc system. Detailed protocol of FokI-(d)pAgo–mediated target cleavage is provided in Supplementary methods section. The target plasmid with different spacers lengths, protein and guide concentrations, guide combinations, guide lengths, type of guide, cleavage time points, cleavage temperatures, and buffer and salt concentrations were modified for the corresponding experiments.

### Cleavage site identification of PNFP editors

Cleavage site identification was conducted as described earlier by Marsic *et al.* (43); the targets cloned into modified pMRS plasmid were linearized with *Bsa*I, purified, and invaded by *γ*PNA1&2. The PNA-invaded templates were further subjected to cleavage using two guide DNAs (both guide DNA1 and guide DNA2) complexed FokI-(d)CbAgo and FokI-(d)KmAgo independently and single guide DNA (either guide DNA1 or guide DNA2) complexed FokI-(d)CbAgo and FokI-(d)KmAgo independently. The PNA invasion and cleavage reaction conditions were employed as described earlier in the methods section. Cleavage products were separated on 1.2% agarose gel by gel electrophoresis. The released top and bottom fragments were eluted independently using QIAquick gel extraction kit (Qiagen - Cat. no. 28706) and subjected to Sanger sequencing with 1444 reverse and 1442 forward primers, respectively (Table S4). The Sanger sequencing reads were analyzed using SnapGene viewer.

## Data availability

The data from this article are available in the article and supporting information.

## Supporting information

This article contains supporting information.

## Conflicts of interest

Authors have a pending patent application on the PNFP editors and their diverse uses and applications in diverse organisms.
