# Supplementary material for: Fusion of FokI and catalytically inactive prokaryotic Argonautes enables site-specific programmable DNA cleavage
Source: J Biol Chem. 2024 Aug 28;300(9):107720. doi: 10.1016/j.jbc.2024.107720 (PMC11421335; doi:10.1016/j.jbc.2024.107720)
Supplement: Supporting information [file mmc1.pdf]

Supporting information

Fusion of FokI and catalytically inactive prokaryotic Argonautes enables site-specific programmable DNA cleavage

Qiaochu Wang[1], Gundra Sivakrishna Rao [1], Tin Marsic[1], Rashid Aman[1], and Magdy Mahfouz[1] *

[1]*Laboratory for Genome Engineering and Synthetic Biology, Division of Biological Sciences, 4700 King Abdullah University of Science and Technology, Thuwal 23955-6900, Saudi Arabia.*

***Correspondence: Magdy M. Mahfouz (magdy.mahfouz@kaust.edu.sa)**

Supplementary figures

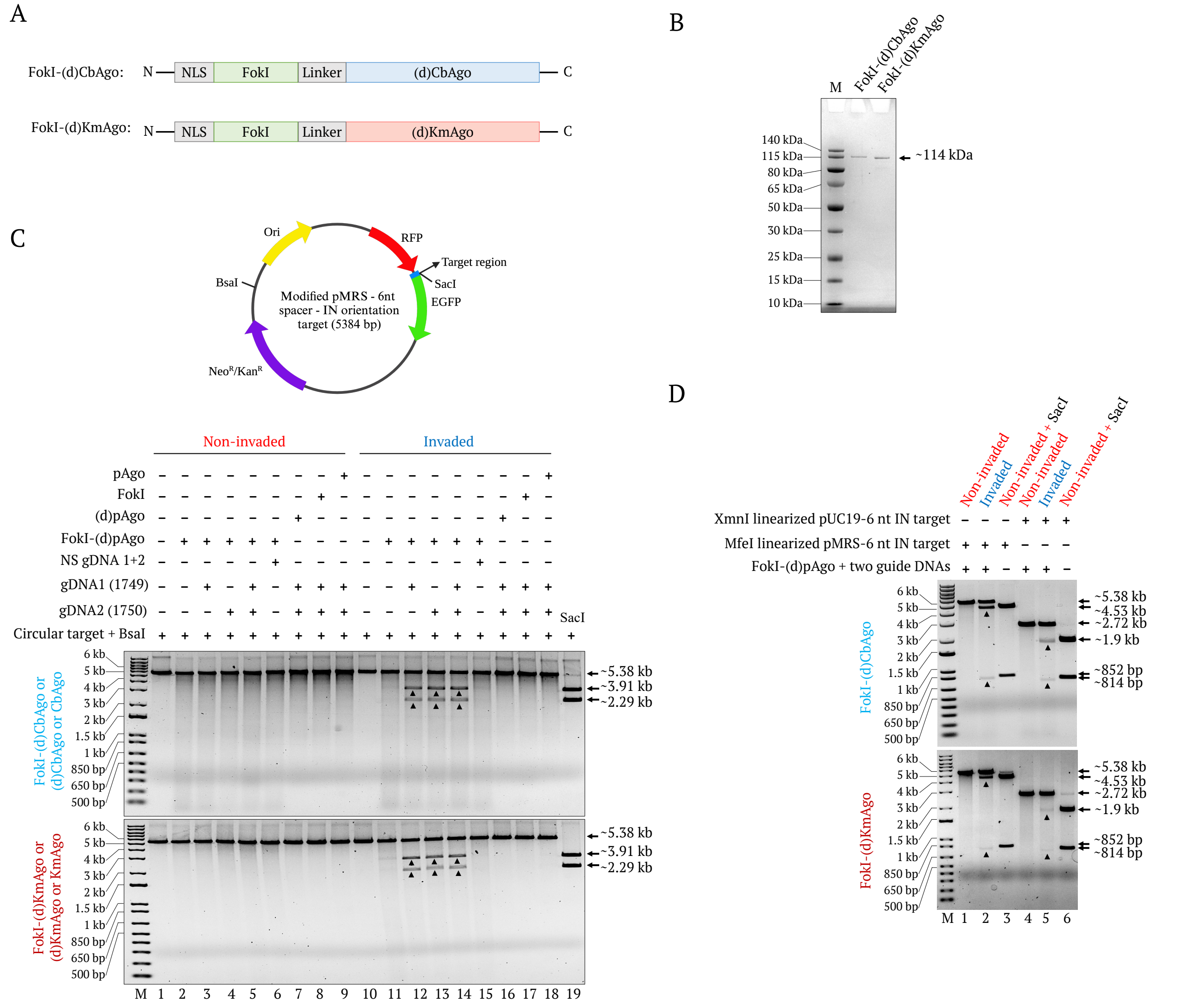

Figure S1. Proof of concept for FokI-(d)pAgo-mediated cleavage of different target plasmids. **(a)** Structure of the FokI-(d)CbAgo and FokI-(d)KmAgo fusion proteins. **(b)** SDS-PAGE analysis of FokI-(d)CbAgo and FokI-(d)KmAgo after size exclusion chromatography. M represents the pre-stained protein ladder (Thermo Scientific^{TM} - 26616). **(c)** Top, diagram of the circular, modified pMRS-6nt spacer - IN orientation target. Bottom, gel images showing the FokI-(d)CbAgo- (top) and FokI-(d)KmAgo- (bottom) mediated cleavage of the circular, non-invaded (Lanes 1–9) and γPNA1&2-invaded (Lanes 10–15) target following *Bsa*I restriction digestion. FokI-(d)pAgo target cleavage was tested using no guides, single specific guides, a pair of specific guides, and a pair of non-specific guides. Reactions including (d)pAgo, FokI, or intact pAgo only for cleavage in the presence of two specific guides were included as controls (Lanes 7–9 [non-invaded] and 16–18 [γPNA1&2-invaded], respectively). In the gel picture the FokI-(d)pAgo is either FokI-(d)CbAgo or

FokI-(d)KmAgo, the (d)pAgo is either (d)CbAgo or (d)KmAgo, and the pAgo is either CbAgo or KmAgo. The *Sac*I restriction enzyme size control is shown in Lane 19. Lane M, 1-kb plus DNA marker. (**d**) FokI-(d)pAgo-mediated cleavage of different plasmids. Gel images showing the FokI-(d)CbAgo- (top) and FokI-(d)KmAgo- (bottom) mediated cleavage of the non-invaded (Lane 1 - MfeI linearized pMRS-6nt IN target and Lane 4 - XmnI linearized pUC19-6nt IN target) and γPNA1&2-invaded (Lane 2 - MfeI linearized pMRS-6nt IN target and Lane 5 - XmnI linearized pUC19-6nt IN target) targets using two guides. The *Sac*I restriction enzyme size controls are shown in Lane 3 (MfeI linearized pMRS-6nt IN target) and Lane 6 (XmnI linearized pUC19-6nt IN target). Lane M, 1-kb plus DNA marker.

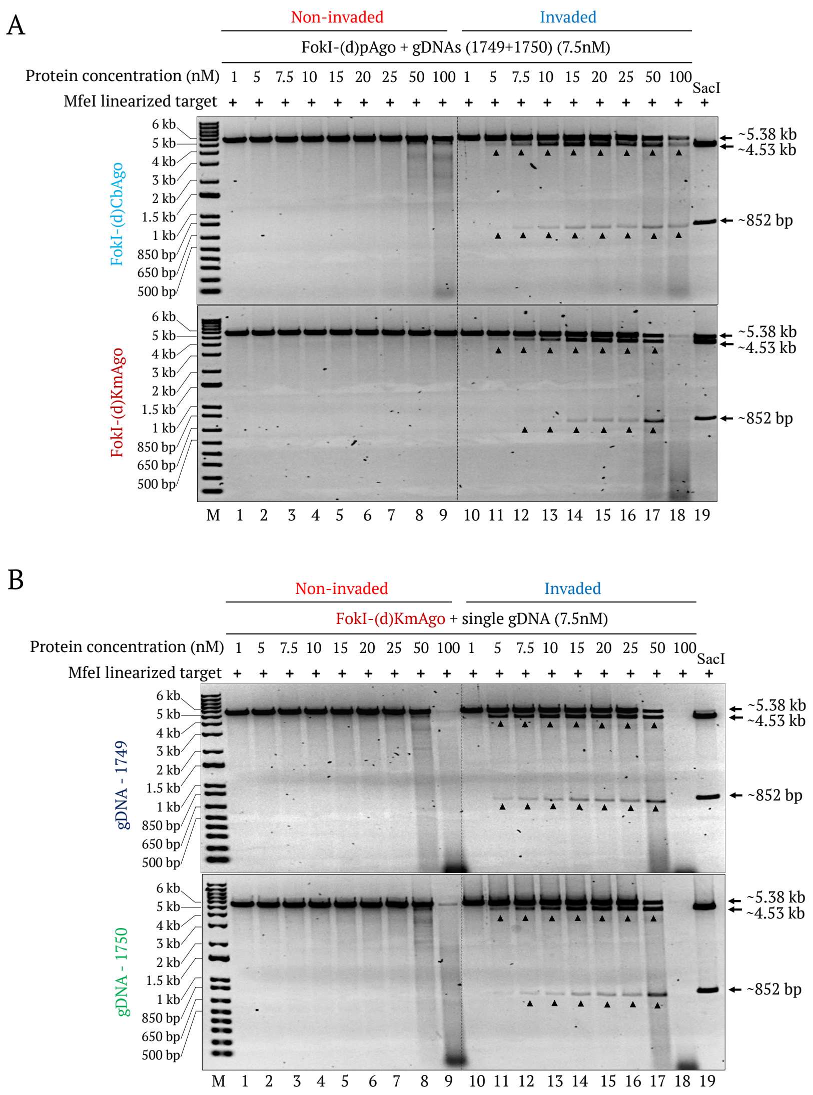

Figure S2. FokI-(d)pAgo protein concentration titration assay. **(a)** Gel images showing FokI-(d)CbAgo- (top) or FokI-(d)KmAgo- (bottom) mediated cleavage of the *Mfe*I-linearized, non-invaded (Lanes 1–9) or γPNA1&2-invaded (Lanes 10–18) modified pMRS-6nt spacer - IN orientation target. FokI-(d)pAgo was added at concentrations of 1 nM, 5 nM, 7.5 nM, 10 nM, 15 nM, 20 nM, 25 nM, 50 nM, and 100 nM and tested using two specific guides (7.5 nM each). The *Sac*I restriction enzyme size control is shown in Lane 19. Lane M, 1-kb plus DNA marker. **(b)** Gel images showing the FokI-(d)KmAgo-mediated cleavage of the *Mfe*I-linearized, non-invaded (Lanes 1–9) or γPNA1&2-invaded (Lanes 10–18) modified pMRS-6nt spacer - IN orientation target using only one gDNA. FokI-(d)KmAgo was added a concentrations of 1 nM, 5 nM, 7.5 nM, 10 nM, 15 nM, 20 nM, 25 nM, 50 nM, or 100 nM and tested using guide DNA1 only (upper gel) or guide DNA2 only (bottom gel). The *Sac*I restriction enzyme size control is shown in Lane 19. Lane M, 1-kb plus DNA marker.

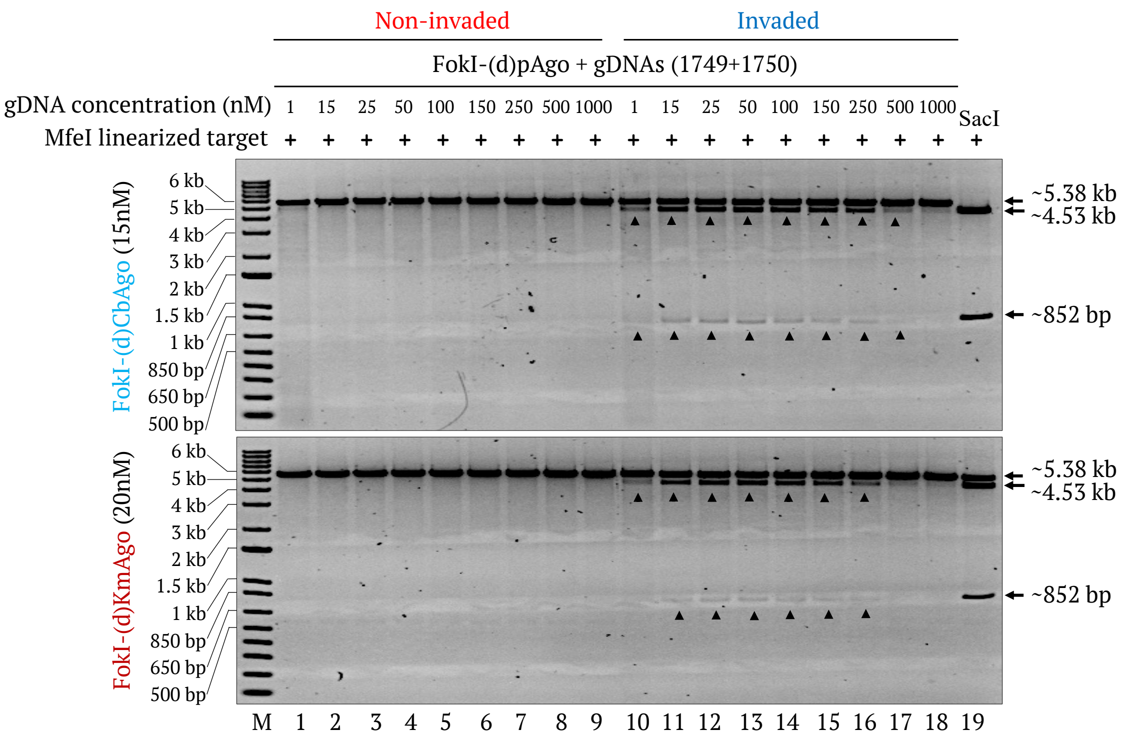

Figure S3. Guide DNA concentration titration assay. Gel images showing the FokI-(d)CbAgo- (top) or FokI-(d)KmAgo- (bottom) mediated cleavage of the *Mfe*I-linearized, non-invaded (Lanes 1–9) or γPNA1&2-invaded (Lanes 10–18) modified pMRS-6nt spacer - IN orientation target. In this experiment, 15 nM FokI-(d)CbAgo or 20 nM FokI-(d)KmAgo was preloaded with different concentrations (1 nM, 15 nM, 25 nM, 50 nM, 100 nM, 150 nM, 250 nM, 500 nM, or 1000 nM) of guide DNAs. The *Sac*I restriction enzyme size control is shown in Lane 19. Lane M, 1-kb plus DNA marker.

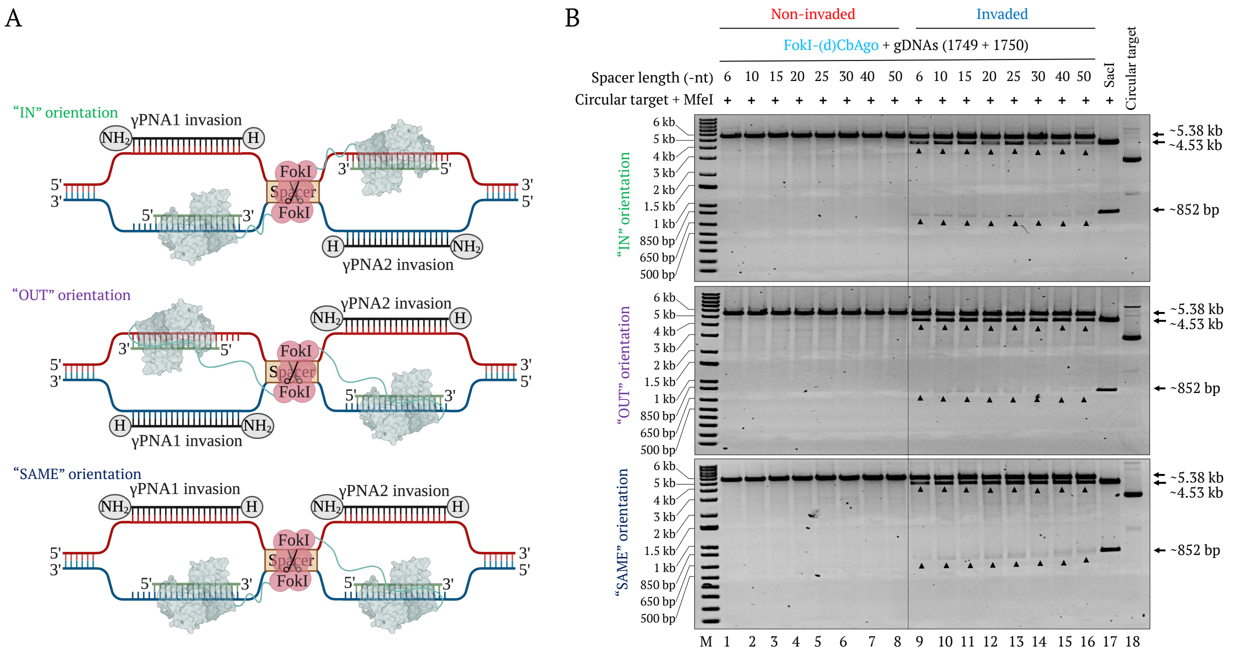

Figure S4. Effect of spacer length and guide orientation on FokI-(d)CbAgo-mediated cleavage of circular dsDNA. **(a)** Diagrams showing the different orientations of the 3' end of the guide (IN, top; OUT, middle; and SAME, bottom). Guide orientation alters the FokI-(d)CbAgo protein binding orientation. Different spacer lengths were designed in each target plasmid. **(b)** Gel images showing the FokI-(d)CbAgo-mediated cleavage of circular, γPNA1&2-invaded, pMRS, "IN" orientation (top), "OUT" orientation (middle), and "SAME" orientation (bottom) targets. Circular, non-invaded (Lanes 1–8) and γPNA1&2-invaded (Lanes 9–16) targets with different spacer lengths (6, 10, 15, 20, 25, 30, 40, or 50 nt) and in different orientations were cleaved with FokI-(d)CbAgo pre-loaded with specific guide DNAs following *Mfe*I restriction digestion. The *Sac*I restriction enzyme size control and undigested plasmid control are shown in Lanes 17 and 18, respectively. Lane M, 1-kb plus DNA marker.

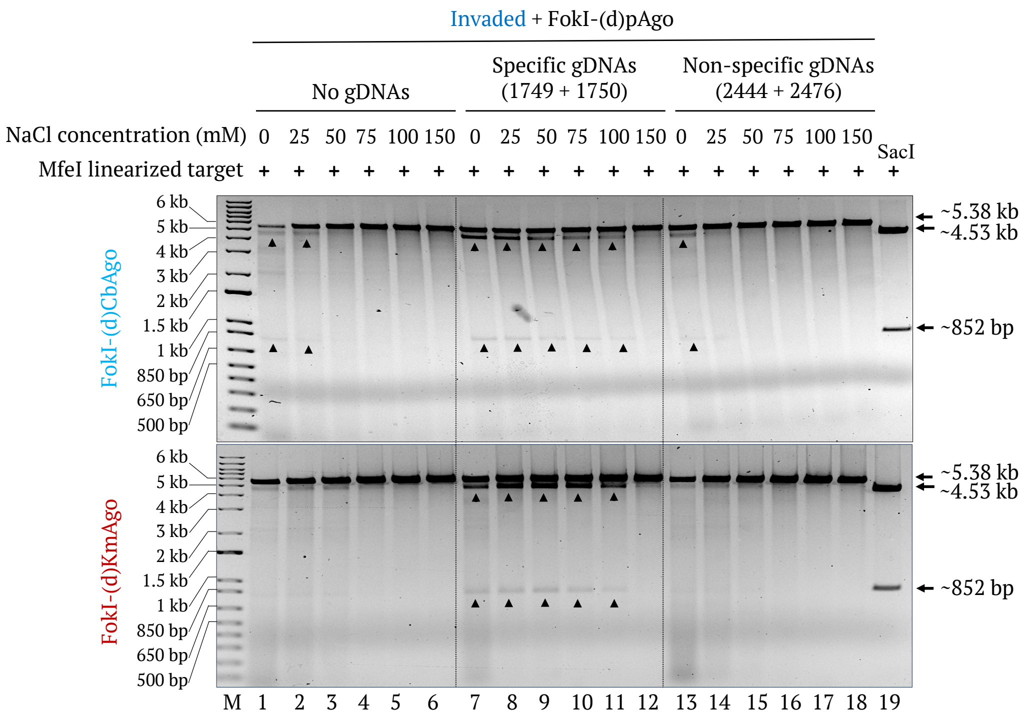

Figure S5. Effect of NaCl concentration on the specificity of FokI-(d)pAgo-mediated dsDNA cleavage. Gel images showing the FokI-(d)CbAgo- (top) or FokI-(d)KmAgo- (bottom) mediated cleavage of the *Mfe*I-linearized, γPNA1&2-invaded, modified pMRS-6nt spacer - IN orientation target. Cleavage reactions were conducted using no guides (Lanes 1–6), a pair of specific guides (Lanes 7–12), or a pair of non-specific guides (Lanes 13–18), and different concentrations of NaCl (0, 25, 50, 75, 100, or 150 nM). The *Sac*I restriction enzyme size control is shown in Lane 19. Lane M, 1-kb plus DNA marker.

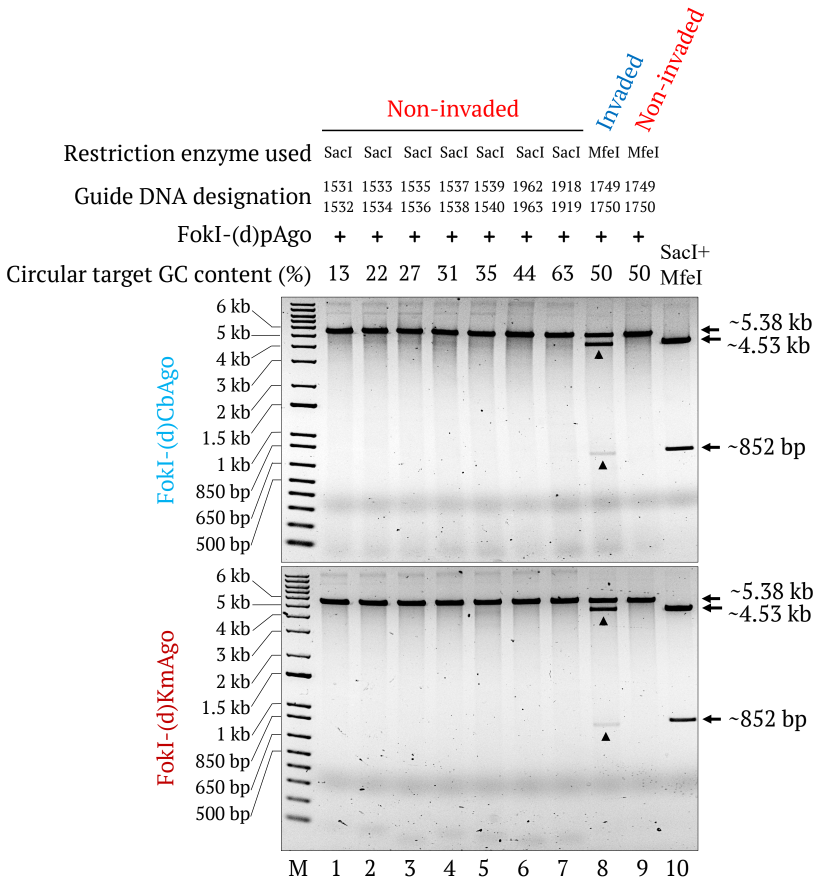

Figure S6. Effect of GC content on FokI-(d)pAgo-mediated cleavage of circular dsDNA. Gel images showing the FokI-(d)CbAgo- (top) or FokI-(d)KmAgo- (bottom) mediated cleavage of a non-invaded, circular, modified pMRS-6nt spacer - IN orientation target. The circular plasmid was incubated with FokI-(d)pAgo pre-loaded with different pairs of guide DNAs that can bind to regions with GC contents of 13%, 22%, 27%, 31%, 35%, 44%, or 63% (Lanes 1–7) following *Sac*I restriction enzyme digestion. A γPNA1&2-invaded and non-invaded, circular target was cleaved using FokI-(d)pAgo pre-loaded with a pair of guide DNAs that can bind to a region with 50% GC content (Lanes 8 and 9, respectively) following *Mfe*I restriction enzyme digestion. The *Sac*I + *Mfe*I restriction enzyme size control is shown in Lane 10. Lane M, 1-kb plus DNA marker.

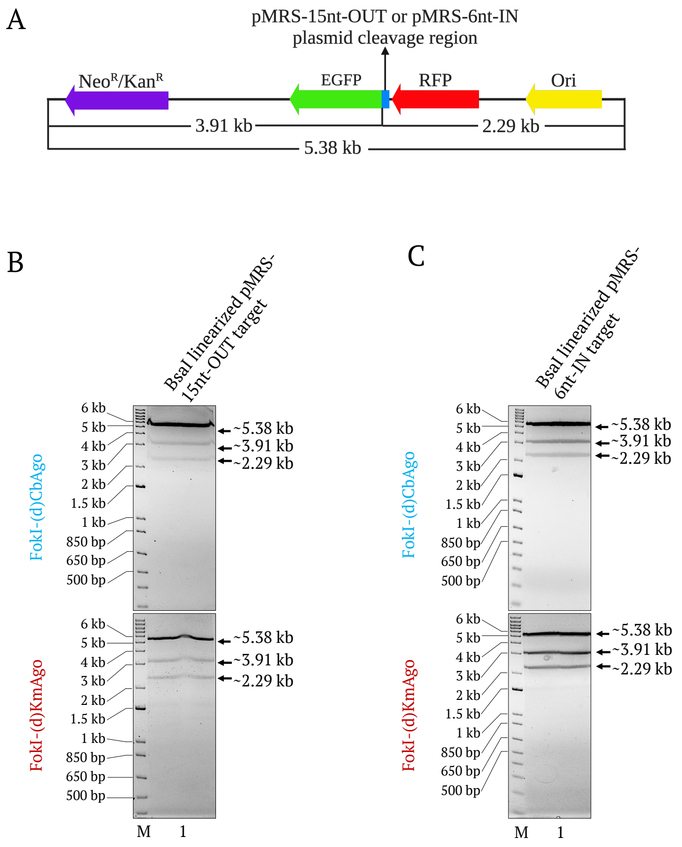

Figure S7. FokI-(d)pAgo mediated cleavage of different pMRS targets. **(a)** BsaI linearized pMRS plasmid map showing the cleavage position and the expected fragment sizes after FokI-(d)pAgo mediated cleavage. **(b)** FokI-(d)CbAgo (top gel) and FokI-(d)KmAgo (bottom gel) mediated cleavage of γPNA1&2-invaded, modified pMRS-15nt spacer - OUT orientation target (Lane 1). **(c)** FokI-(d)CbAgo (top gel) and FokI-(d)KmAgo (bottom gel) mediated cleavage of γPNA1&2-invaded, modified pMRS-6nt spacer - IN orientation target (Lane 1). All the released fragments are subjected to Sanger sequencing using corresponding primers. Lane M, 1-kb plus DNA marker.

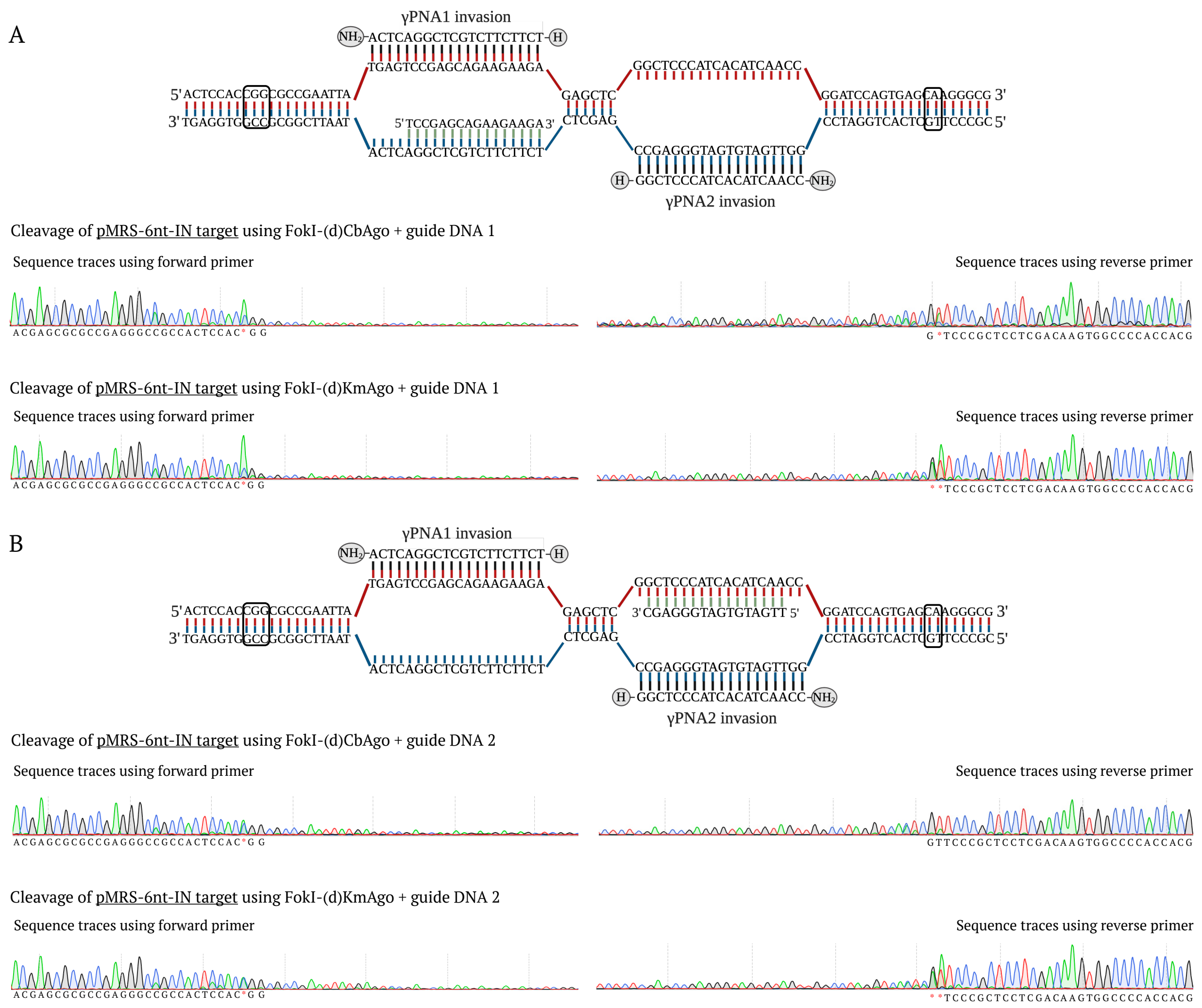

Figure S8. Cleavage site identification of FokI-(d)pAgo on pMRS-6 nt-IN target. **(a)** *Bsa*I-linearized, γPNA1&2-invaded, modified pMRS-6nt spacer - IN orientation target bound with guide DNA1 represented on top. Sanger sequencing reads of FokI-(d)CbAgo and FokI-(d)KmAgo cleaved products using forward primer and reverse primer represented below. **(c)** *Bsa*I-linearized, γPNA1&2-invaded, modified pMRS-6nt spacer - IN orientation target bound with guide DNA2 represented on top. Sanger sequencing reads of FokI-(d)CbAgo and FokI-(d)KmAgo cleaved products using forward primer and reverse primer represented below. Box indicate the approximate cleavage positions of FokI-(d)CbAgo and FokI-(d)KmAgo; asterisk is the mismatched nucleotide read after Sanger sequencing.

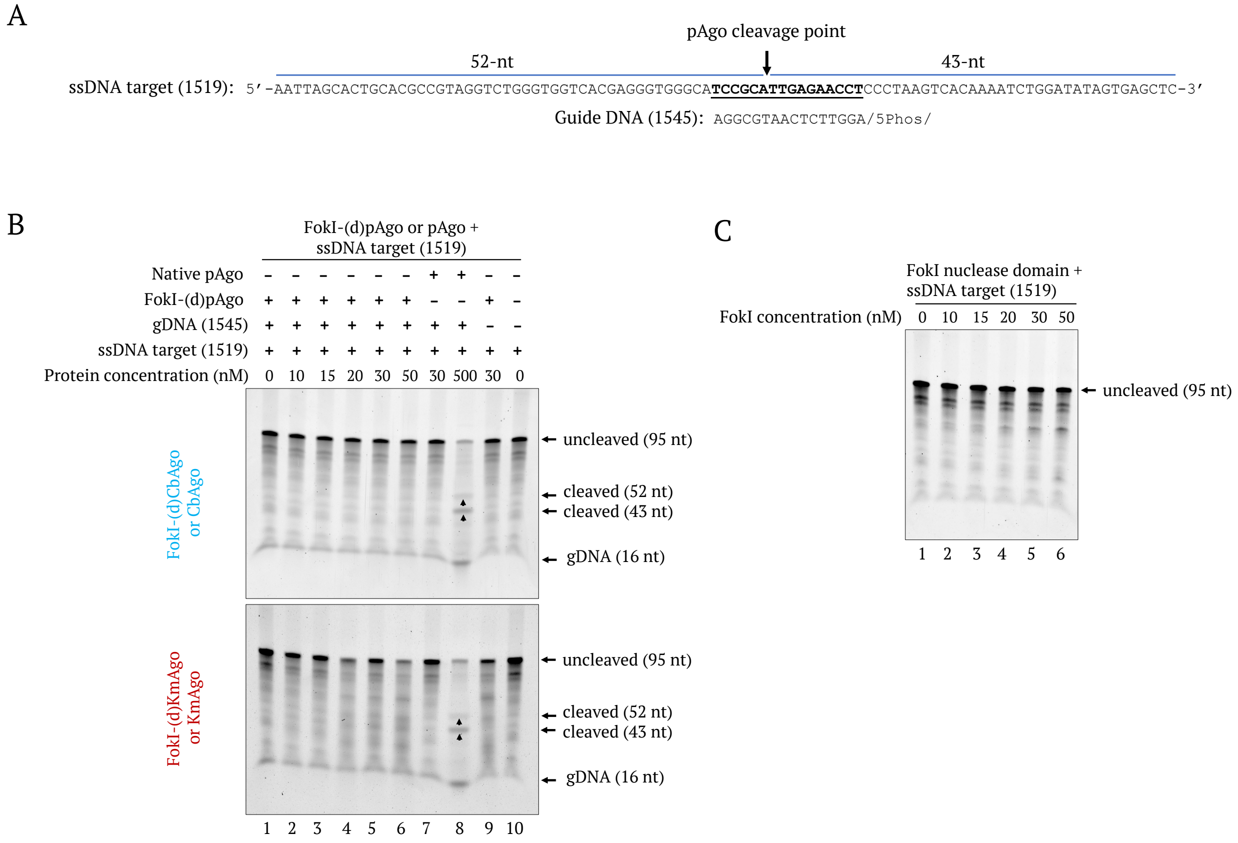

Figure S9. FokI-(d)pAgo activity on ssDNA. **(a)** Sequence of ssDNA and corresponding guide DNA binding site. Arrow showing the native pAgo cleavage point. **(b)** Gel images showing the FokI-(d)CbAgo- (top) or FokI-(d)KmAgo- (bottom) mediated cleavage of a ssDNA target using different protein concentrations. ssDNA was incubated with guide DNA loaded FokI-(d)CbAgo- (top) or FokI-(d)KmAgo- (bottom) at 0 nM, 10 nM, 15nM, 20nM, 30nM and 50nM concentrations (Lanes 1-6). Lane 7 is ssDNA incubated with 30nM of native CbAgo- (top) or native KmAgo- (bottom) loaded with corresponding gDNA. Lane 8 is ssDNA incubated with 500nM of native CbAgo- (top) or native KmAgo- (bottom) loaded with corresponding gDNA as a size control. Lane 9 is a no guide control; ssDNA incubated with 30nM of FokI-(d)CbAgo- (top) or FokI-(d)KmAgo- (bottom). Lane 10 is only ssDNA as negative control. **(c)** Fok1 nuclease domain activity on ssDNA. ssDNA target incubated with different concentrations (0 nM – 50 nM) of Fok1 nuclease protein (Lanes 1-6).

Supplementary files

1) Sequence of expression vectors for FokI-(d)pAgo purification used in this study

A. NLS-FokI-(d)CbAgo-Twin-Strep-SUMO

tcatgaccaaaatcccttaacgtgagttttcgttccactgagcgtcagaccccgtagaaaagatcaaagg
atcttcttgagatcctttttttctgcgcgtaatctgctgcttgcaaacaaaaaaaccaccgctaccagcg
gtggtttgtttgccggatcaagagctaccaactctttttccgaaggtaactggcttcagcagagcgcaga
taccaaatactgtccttctagtgtagccgtagttaggccaccacttcaagaactctgtagcaccgcctac
atacctcgctctgctaatcctgttaccagtggctgctgccagtggcgataagtcgtgtcttaccgggttg
gactcaagacgatagttaccggataaggcgcagcggtcgggctgaacggggggttcgtgcacacagccca
gcttggagcgaacgacctacaccgaactgagatacctacagcgtgagctatgagaaagcgccacgcttcc
cgaagggagaaaggcggacaggtatccggtaagcggcagggtcggaacaggagagcgcacgagggagctt
ccagggggaaacgcctggtatctttatagtcctgtcgggtttcgccacctctgacttgagcgtcgatttt
tgtgatgctcgtcaggggggcggagcctatggaaaaacgccagcaacgcggcctttttacggttcctggc
cttttgctggccttttgctcacatgttctttcctgcgttatcccctgattctgtggataaccgtattacc
gcctttgagtgagctgataccgctcgccgcagccgaacgaccgagcgcagcgagtcagtgagcgaggaag
cggaagagcgcctgatgcggtattttctccttacgcatctgtgcggtatttcacaccgcaatggtgcact
ctcagtacaatctgctctgatgccgcatagttaagccagtatacactccgctatcgctacgtgactgggt
catggctgcgccccgacacccgccaacacccgctgacgcgccctgacgggcttgtctgctcccggcatcc
gcttacagacaagctgtgaccgtctccgggagctgcatgtgtcagaggttttcaccgtcatcaccgaaac
gcgcgaggcagctgcggtaaagctcatcagcgtggtcgtgaagcgattcacagatgtctgcctgttcatc
cgcgtccagctcgttgagtttctccagaagcgttaatgtctggcttctgataaagcgggccatgttaagg
gcggtttttttcctgtttggtcactgatgcctccgtgtaagggggatttctgttcatgggggtaatgatac
cgatgaaacgagagaggatgctcacgatacgggttactgatgatgaacatgcccggttactggaacgttg
tgagggtaaacaactggcggtatggatgcggcgggaccagagaaaaatcactcagggtcaatgccagcgc
ttcgttaatacagatgtaggtgttccacagggtagccagcagcatcctgcgatgcagatccggaacataa
tggtgcagggcgctgacttccgcgtttccagactttacgaaacacggaaaccgaagaccattcatgttgt
tgctcaggtcgcagacgttttgcagcagcagtcgcttcacgttcgctcgcgtatcggtgattcattctgc
taaccagtaaggcaaccccgccagcctagccgggtcctcaacgacaggagcacgatcatgcgcacccgtg
gccaggacccaacgctgcccgagatgcgccgcgtgcggctgctggagatggcggacgcgatggatatgtt
ctgccaagggttggtttgcgcattcacagttctccgcaagaattgattggctccaattcttggagtggtg
aatccgttagcgaggtgccgccggcttccattcaggtcgaggtggcccggctccatgcaccgcgacgcaa
cgcggggaggcagacaaggtatagggcggcgcctacaatccatgccaacccgttccatgtgctcgccgag
gcggcataaatcgccgtgacgatcagcggtccaatgatcgaagttaggctggtaagagccgcgagcgatc
cttgaagctgtccctgatggtcgtcatctacctgcctggacagcatggcctgcaacgcgggcatcccgat
gccgccggaagcgagaagaatcataatggggaaggccatccagcctcgcgtcgcgaacgccagcaagacg
tagcccagcgcgtcggccgccatgccggcgataatggcctgcttctcgccgaaacgtttggtggcgggac
cagtgacgaaggcttgagcgagggcgtgcaagattccgaataccgcaagcgacaggccgatcatcgtcgc
gctccagcgaaagcggtcctcgccgaaaatgacccagagcgctgccggcacctgtcctacgagttgcatg
ataaagaagacagtcataagtgcggcgacgatagtcatgccccgcgcccaccggaaggagctgactgggt
tgaaggctctcaagggcatcggtcgagatcccggtgcctaatgagtgagctaacttacattaattgcgtt
gcgctcactgcccgctttccagtcgggaaacctgtcgtgccagctgcattaatgaatcggccaacgcgcg
gggagaggcggtttgcgtattgggcgccagggtggtttttcttttcaccagtgagacgggcaacagctga
ttgcccttcaccgcctggccctgagagagttgcagcaagcggtccacgctggtttgccccagcaggcgaa
aatcctgtttgatggtggttaacggcgggatataacatgagctgtcttcggtatcgtcgtatcccactac
cgagatatccgcaccaacgcgcagcccggactcggtaatggcgcgcattgcgcccagcgccatctgatcg
ttggcaaccagcatcgcagtgggaacgatgccctcattcagcatttgcatggtttgttgaaaaccggaca
tggcactccagtcgccttcccgttccgctatcggctgaatttgattgcgagtgagatatttatgccagcc

agccagacgcagacgcgccgagacagaacttaatgggcccgctaacagcgcgatttgctggtgacccaat
gcgaccagatgctccacgcccagtcgcgtaccgtcttcatgggagaaaataatactgttgatgggtgtct
ggtcagagacatcaagaaataacgccggaacattagtgcaggcagcttccacagcaatggcatcctggtc
atccagcggatagttaatgatcagcccactgacgcgttgcgcgagaagattgtgcaccgccgctttacag
gcttcgacgccgcttcgttctaccatcgacaccaccacgctggcacccagttgatcggcgcgagatttaa
tcgccgcgacaatttgcgacggcgcgtgcagggccagactggaggtggcaacgccaatcagcaacgactg
tttgcccgccagttgttgtgccacgcggttgggaatgtaattcagctccgccatcgccgcttccactttt
tcccgcgttttcgcagaaacgtggctggcctggttcaccacgcgggaaacggtctgataagagacaccgg
catactctgcgacatcgtataacgttactggtttcacattcaccaccctgaattgactctcttccgggcg
ctatcatgccataccgcgaaaggttttgcgccattcgatggtgtccgggatctcgacgctctcccttatg
cgactcctgcattaggaagcagcccagtagtaggttgaggccgttgagcaccgccgccgcaaggaatggt
gcatgcaaggagatggcgcccaacagtcccccggccacggggcctgccaccatacccacgccgaaacaag
cgctcatgagcccgaagtggcgagcccgatcttccccatcggtgatgtcggcgatataggcgccagcaac
cgcacctgtggcgccggtgatgccggccacgatgcgtccggcgtagaggatcgagatctcgatcccgcga
aattaatacgactcactataggggaattgtgagcggataacaattcccctctagaaataattttgtttaa
ctttaagaaggagatataccatgggcagcagccatcatcatcatcatcacagcagcggcctggtgccgcg
cggcagccatatggctagctggagccatccgcagtttgaaaaaggtggtggtagcggtggtggttcaggt
ggtagtgcatggtcacaccctcagtttgagaaaatgtcggactcagaagtcaatcaagaagctaagccag
aggtcaagccagaagtcaagcctgagactcacatcaatttaaaggtgtccgatggatcttcagagatctt
cttcaagatcaaaaagaccactcctttaagaaggctgatggaagcgttcgctaaaagacagggtaaggaa
atggactccttaagattcttgtacgacggtattagaatccaagctgatcagacccctgaagatttggaca
tggaggataacgatattattgaggctcacagagaacagattggtggaTCCATGGCTCCCAAAAAAAAAAG
GAAGGTAGGAATCCACCGTGGTGTTCCGGGCGGTAGCATGGGTTCGCAACTGGTTAAAAGCGAATTAGAG
GAGAAGAAATCCGAGCTGCGTCATAAACTTAAATACGTTCCGCATGAATACATTGAACTGATCGAGATTG
CTCGCAACAGCACCCAAGATCGTATTTTGGAAATGAAAGTGATGGAATTTTTTATGAAAGTCTATGGTTA
CCGTGGTAAACACCTGGGCGGAAGCCGTAAACCGGATGGTGCGATTTACACCGTTGGTTCGCCGATCGAC
TATGGTGTAATTGTCGATACCAAAGCCTATTCTGGTGGCTATAACTTACCGATCGGCCAAGCGGATGAAA
TGCAGCGTTATGTTGAGGAAAACCAAACCCGCAATAAGCACATCAATCCAAATGAATGGTGGAAGGTGTA
CCCGAGCAGCGTAACTGAGTTCAAATTCTTGTTCGTGAGCGGCCATTTCAAGGGCAATTATAAAGCGCAA
CTGACGCGTCTCAATCATATCACGAACTGCAACGGCGCGGTTCTGAGCGTTGAAGAATTGCTGATTGGTG
GTGAAATGATTAAAGCGGGCACTCTAACCTTGGAAGAGGTGAGGCGTAAGTTCAATAACGGTGAAATCAA
TTTCTCTGGAGGCTCTTCCGGCGGCTCCTCTGGTAGTGAAACGCCGGGCACCAGCGAATCTGCGACCCCT
GAGTCCAGCGGAGGTAGCTCTGGTGGCTCCAGTATGAATAATTTGACGTTTGAGGCTTTTGAAGGTATCG
GTCAACTGAACGAGCTGAACTTCTACAAGTACCGCTTAATCGGCAAGGGTCAGATCGATAATGTTCATCA
GGCTATCTGGAGCGTTAAATACAAGTTGCAAGCTAATAACTTCTTTAAACCCGTTTTCGTCAAAGGTGAG
ATCCTGTACAGCCTGGATGAATTGAAGGTGATACCGGAGTTCGAGAACGTGGAGGTGATCCTGGACGGCA
ATATTATTTTGTCTATTTCAGAGAACACCGATATTTATAAGGATGTGATCGTGTTTTATATTAACAACGC
ACTGAAAAACATCAAGGACATCACCAACTACCGTAAATACATCACCAAGAACACCGACGAGATTATCTGC
AAGTCCATCCTTACGACCAACCTGAAGTACCAGTATATGAAAAGCGAAAAAGGTTTCAAACTGCAACGTA
AATTTAAGATTAGCCCGGTGGTCTTTCGTAACGGTAAGGTCATCCTCTACCTTAACTGCAGCAGCGATTT
TTCCACCGACAAATCGATCTACGAAATGCTGAACAACGGGTTAGACGTCGTGGGCTTACAGGTAAAAAAC
CGTTGGACCAATAGCAATGGCAACATCTTCATTGAAGAGGTGCTGGACAAATCAATTTCTGAGCCGGGTA
CGTCCGGTAAACTCGGCCAGTCGCTGATTGATTACTATATCAACGGCAACCAGAAATACCGTGTGGAAAA
GTTCACCGATGAGGACAAGAAGGCTAAGGTTATCAAGGCGAAAATTAAGAACAAGACCTATAATTATATC
CCGCAGGCCCTGACGCCGGTAATCACGAGAGAGTACCTGTCCCACACCGATAAAAAGTTCAGCAAACAAA
TCGAGAATGTGATCAAAATGGATATGAACTATCGCTATCAGACCCTCAAATCTTTTGTGGAGGACATCGG
TGTCATCAAAGAGCTGAACAACCTTCACTTTAAGAACCAGTATTACACCAATTTCGACTTCATGGGCTTC
GAATCTGGCATCTTGGAAGAGCCGGTGCTGATGGGTGCGAACGGTAAAATTAAGGATAAAAAGCAGATTT
TTATCAACGGCTTCTTTAAAAACCCGAAAGAAAACGTTAAGTTTGGTGTTCTCTACCCGGAGGGCTGCAT
GGAAAACGCCCAGAGCATCGCTCGCAGCATCTTGGACTTTGCGACCGCGGGTAAATACAACAAGCAAGAG
AATAAGTACATAAGCAAGAACCTGATGAACATCGGCTTCAAGCCTTCCGAATGTATTTTCGAGAGCTATA
AGCTGGGTGACATCACGGAATACAAGGCTACTGCCAGAAAGTTGAAAGAGCACGAAAAGGTTGGCTTCGT

GATCGCGGTGATTCCGGACATGAATGAATCCGAGGTGGAGAATCCGTATAACCCATTTAAGAAGGTTTGG
GCAAAACTGAACATCCCGAGCCAGATGATTACCCTGAAAACCACTGAGAAATTCAAAAACATTGTCGATA
AGTCCGGTCTGTATTACCTGCATAATATTGCACTGAACATCCTGGGCAAGATTGGAGGCATTCCGTGGAT
TATAAAGGACATGCCGGGTAACATCGACTGTTTTATCGGCCTGGCAGTTGGCACCCGCGAGAAAGGCATC
CACTTTCCGGCATGTAGCGTGTTGTTTGACAAGTATGGCAAACTGATTAACTACTACAAACCGACCATTC
CGCAAAGCGGTGAAAAAATTGCGGAAACCATTCTGCAAGAAATCTTCGACAACGTGTTGATTTCCTACAA
AGAGGAAAATGGCGAGTATCCAAAAAATATTGTAATCCACCGCGCGGGTTTCAGCCGTGAGAACATTGAC
TGGTATAAAGAATATTTTGACAAAAAAGGTATTAAGTTCAACATTATCGAGGTGAAGAAAAACATTCCGG
TGAAGATCGCGAAAGTTGTGGGCAGCAATATCTGCAATCCGATTAAGGGCAGCTATGTATTGAAGAACGA
TAAAGCCTTTATTGTTACCACCGATATTAAGGACGGTGTTGCGAGCCCGAACCCGCTGAAAATCGAGAAG
ACCTACGGTGACGTCGAAATGAAGTCGATTTTGGAGCAGATTTACAGCCTGTCTCAGATCCACGTTGGTA
GTACTAAATCTCTGCGCCTGCCGATTACCACCGGCTATGCCGATAAGATTTGCAAGGCGATCGAATACAT
CCCGCAGGGTGTGGTGGACAATCGTTTGTTCTTCCTGtaagcggccgcactcgaggcccgaaaggaagct
gagttggctgctgccaccgctgagcaataactagcataaccccttggggcctctaaacgggtcttgaggg
gtttttttgctgaaaggaggaactatatccggatatcccgcaagaggcccggcagtaccggcataaccaag
cctatgcctacagcatccagggtgacggtgccgaggatgacgatgagcgcattgttagatttcatacacg
gtgcctgactgcgttagcaatttaactgtgataaactaccgcattaaagcttatcgatgataagctgtca
aacatgagaattcttgaagacgaaagggcctcgtgatacgcctatttttataggttaatgtcatgataat
aatggtttcttagacgtcaggtggcacttttcggggaaatgtgcgcggaacccctatttgtttatttttc
taaatacattcaaatatgtatccgctcatgagacaataaccctgataaatgcttcaataatattgaaaaa
ggaagagtatgagtattcaacatttccgtgtcgcccttattcccttttttgcggcattttgccttcctgt
ttttgctcacccagaaacgctggtgaaagtaaaagatgctgaagatcagttgggtgcacgagtgggttac
atcgaactggatctcaacagcggtaagatccttgagagttttcgccccgaagaacgttttccaatgatga
gcacttttaaagttctgctatgtggcgcggtattatcccgtgttgacgccgggcaagagcaactcggtcg
ccgcatacactattctcagaatgacttggttgagtactcaccagtcacagaaaagcatcttacggatggc
atgacagtaagagaattatgcagtgctgccataaccatgagtgataacactgcggccaacttacttctga
caacgatcggaggaccgaaggagctaaccgcttttttgcacaacatgggggatcatgtaactcgccttga
tcgttgggaaccggagctgaatgaagccataccaaacgacgagcgtgacaccacgatgcctgcagcaatg
gcaacaacgttgcgcaaactattaactggcgaactacttactctagcttcccggcaacaattaatagact
ggatggaggcggataaagttgcaggaccacttctgcgctcggcccttccggctggctggtttattgctga
taaatctggagccggtgagcgtgggtctcgcggtatcattgcagcactggggccagatggtaagccctcc
cgtatcgtagttatctacacgacggggagtcaggcaactatggatgaacgaaatagacagatcgctgaga
taggtgcctcactgattaagcattggtaactgtcagaccaagtttactcatatatactttagattgattt
aaaacttcatttttaatttaaaaggatctaggtgaagatcctttttgataatc

The uppercase, underlined sequence encodes NLS-FokI-(d)CbAgo.

B. pET28a-6Xhis-HRV3C-NLS-FokI-(d)KmAgo

tggcgaatgggacgcgccctgtagcggcgcattaagcgcggcgggtgtggtggttacgcgcagcgtgacc
gctacacttgccagcgccctagcgcccgctcctttcgctttcttcccttcctttctcgccacgttcgccg
gctttccccgtcaagctctaaatcgggggctccctttagggttccgatttagtgctttacggcacctcga
ccccaaaaaacttgattagggtgatggttcacgtagtgggccatcgccctgatagacggtttttcgccct
ttgacgttggagtccacgttctttaatagtggactcttgttccaaactggaacaacactcaaccctatct
cggtctattcttttgatttataagggattttgccgatttcggcctattggttaaaaaatgagctgattta
acaaaaatttaacgcgaattttaacaaaatattaacgtttacaatttcaggtggcacttttcggggaaat
gtgcgcggaacccctatttgtttatttttctaaatacattcaaatatgtatccgctcatgaattaattct
tagaaaaactcatcgagcatcaaatgaaactgcaatttattcatatcaggattatcaataccatattttt

gaaaaagccgtttctgtaatgaaggagaaaactcaccgaggcagttccataggatggcaagatcctggta
tcggtctgcgattccgactcgtccaacatcaatacaacctattaatttcccctcgtcaaaaataaggtta
tcaagtgagaaatcaccatgagtgacgactgaatccggtgagaatggcaaaagtttatgcatttctttcc
agacttgttcaacaggccagccattacgctcgtcatcaaaatcactcgcatcaaccaaaccgttattcat
tcgtgattgcgcctgagcgagacgaaatacgcgatcgctgttaaaaggacaattacaaacaggaatcgaa
tgcaaccggcgcaggaacactgccagcgcatcaacaatattttcacctgaatcaggatattcttctaata
cctggaatgctgttttcccggggatcgcagtggtgagtaaccatgcatcatcaggagtacggataaaatg
cttgatggtcggaagaggcataaattccgtcagccagtttagtctgaccatctcatctgtaacatcattg
gcaacgctacctttgccatgtttcagaaacaactctggcgcatcgggcttcccatacaatcgatagattg
tcgcacctgattgcccgacattatcgcgagcccatttatacccatataaatcagcatccatgttggaatt
taatcgcggcctagagcaagacgtttcccgttgaatatggctcataacaccccttgtattactgtttatg
taagcagacagttttattgttcatgaccaaaatcccttaacgtgagttttcgttccactgagcgtcagac
cccgtagaaaagatcaaaggatcttcttgagatcctttttttctgcgcgtaatctgctgcttgcaaacaa
aaaaaccaccgctaccagcggtggtttgtttgccggatcaagagctaccaactctttttccgaaggtaac
tggcttcagcagagcgcagataccaaatactgtccttctagtgtagccgtagttaggccaccacttcaag
aactctgtagcaccgcctacatacctcgctctgctaatcctgttaccagtggctgctgccagtggcgata
agtcgtgtcttaccgggttggactcaagacgatagttaccggataaggcgcagcggtcgggctgaacggg
gggttcgtgcacacagcccagcttggagcgaacgacctacaccgaactgagatacctacagcgtgagcta
tgagaaagcgccacgcttcccgaagggagaaaggcggacaggtatccggtaagcggcagggtcggaacag
gagagcgcacgagggagcttccagggggaaacgcctggtatctttatagtcctgtcgggtttcgccacct
ctgacttgagcgtcgatttttgtgatgctcgtcaggggggcggagcctatggaaaaacgccagcaacgcg
gcctttttacggttcctggccttttgctggccttttgctcacatgttctttcctgcgttatcccctgatt
ctgtggataaccgtattaccgcctttgagtgagctgataccgctcgccgcagccgaacgaccgagcgcag
cgagtcagtgagcgaggaagcggaagagcgcctgatgcggtattttctccttacgcatctgtgcggtatt
tcacaccgcatatatggtgcactctcagtacaatctgctctgatgccgcatagttaagccagtatacact
ccgctatcgctacgtgactgggtcatggctgcgccccgacacccgccaacacccgctgacgcgccctgac
gggcttgtctgctcccggcatccgcttacagacaagctgtgaccgtctccgggagctgcatgtgtcagag
gttttcaccgtcatcaccgaaacgcgcgaggcagctgcggtaaagctcatcagcgtggtcgtgaagcgat
tcacagatgtctgcctgttcatccgcgtccagctcgttgagtttctccagaagcgttaatgtctggcttc
tgataaagcgggccatgttaagggcggttttttcctgtttggtcactgatgcctccgtgtaagggggatt
tctgttcatgggggtaatgataccgatgaaacgagagaggatgctcacgatacgggttactgatgatgaa
catgcccggttactggaacgttgtgagggtaaacaactggcggtatggatgcggcgggaccagagaaaaa
tcactcagggtcaatgccagcgcttcgttaatacagatgtaggtgttccacagggtagccagcagcatcc
tgcgatgcagatccggaacataatggtgcagggcgctgacttccgcgtttccagactttacgaaacacgg
aaaccgaagaccattcatgttgttgctcaggtcgcagacgttttgcagcagcagtcgcttcacgttcgct
cgcgtatcggtgattcattctgctaaccagtaaggcaaccccgccagcctagccgggtcctcaacgacag
gagcacgatcatgcgcacccgtggggccgccatgccggcgataatggcctgcttctcgccgaaacgtttg
gtggcgggaccagtgacgaaggcttgagcgagggcgtgcaagattccgaataccgcaagcgacaggccga
tcatcgtcgcgctccagcgaaagcggtcctcgccgaaaatgacccagagcgctgccggcacctgtcctac
gagttgcatgataaagaagacagtcataagtgcggcgacgatagtcatgccccgcgcccaccggaaggag
ctgactgggttgaaggctctcaagggcatcggtcgagatcccggtgcctaatgagtgagctaacttacat
taattgcgttgcgctcactgcccgctttccagtcgggaaacctgtcgtgccagctgcattaatgaatcgg
ccaacgcgcggggagaggcggtttgcgtattgggcgccagggtggtttttcttttcaccagtgagacggg
caacagctgattgcccttcaccgcctggccctgagagagttgcagcaagcggtccacgctggtttgcccc
agcaggcgaaaatcctgtttgatggtggttaacggcgggatataacatgagctgtcttcggtatcgtcgt
atcccactaccgagatatccgcaccaacgcgcagcccggactcggtaatggcgcgcattgcgcccagcgc
catctgatcgttggcaaccagcatcgcagtgggaacgatgccctcattcagcatttgcatggtttgttga
aaaccggacatggcactccagtcgccttcccgttccgctatcggctgaatttgattgcgagtgagatatt
tatgccagccagccagacgcagacgcgccgagacagaacttaatgggcccgctaacagcgcgatttgctg
gtgacccaatgcgaccagatgctccacgcccagtcgcgtaccgtcttcatgggagaaaataatactgttg
atgggtgtctggtcagagacatcaagaaataacgccggaacattagtgcaggcagcttccacagcaatgg
catcctggtcatccagcggatagttaatgatcagcccactgacgcgttgcgcgagaagattgtgcaccgc

cgctttacaggcttcgacgccgcttcgttctaccatcgacaccaccacgctggcacccagttgatcggcg
cgagatttaatcgccgcgacaatttgcgacggcgcgtgcagggccagactggaggtggcaacgccaatca
gcaacgactgtttgcccgccagttgttgtgccacgcggttgggaatgtaattcagctccgccatcgccgc
ttccacttttcccgcgttttcgcagaaacgtggctggcctggttcaccacgcgggaaacggtctgataa
gagacaccggcatactctgcgacatcgtataacgttactggtttcacattcaccaccctgaattgactct
cttccgggcgctatcatgccataccgcgaaaggttttgcgccattcgatggtgtccgggatctcgacgct
ctcccttatgcgactcctgcattaggaagcagcccagtagtaggttgaggccgttgagcaccgccgccgc
aaggaatggtgcatgcaaggagatggcgcccaacagtcccccggccacggggcctgccaccatacccacg
ccgaaacaagcgctcatgagcccgaagtggcgagcccgatcttccccatcggtgatgtcggcgatatagg
cgccagcaaccgcacctgtggcgccggtgatgccggccacgatgcgtccggcgtagaggatcgagatctc
gatcccgcgaaattaatacgactcactataggggaattgtgagcggataacaattcccctctagaaataa
ttttgtttaactttaagaaggagatataccATGCATCACCATCACCATCACACTAGTCCCAAAAAAAAAA
GGAAGGTAGGAATCCACCGCGGTGTGCCGGGTGGTTCGATGGGTTCTCAGCTGGTGAAGAGCGAATTGGA
GGAAAAAAAGTCCGAACTCCGTCACAAACTGAAGTATGTGCCGCACGAATACATCGAGTTGATTGAGATC
GCTCGTAATAGCACCCAGGATCGTATTCTCGAAATGAAGGTGATGGAGTTCTTTATGAAAGTGTATGGTT
ACCGCGGCAAACACCTGGGTGGTAGCCGTAAACCAGATGGTGCGATTTACACCGTTGGTAGTCCGATCGA
CTATGGCGTTATTGTTGATACCAAGGCATATAGCGGTGGCTACAACCTGCCGATAGGCCAGGCCGACGAG
ATGCAGCGTTATGTGGAAGAGAATCAGACCCGCAACAAACACATTAACCCGAATGAGTGGTGGAAAGTTT
ACCCGAGCAGCGTTACCGAGTTCAAATTCCTGTTTGTTTCGGGTCATTTCAAAGGTAACTATAAGGCCCA
ATTGACCCGTTTAAATCATATCACGAACTGCAATGGGGCGGTTCTGTCCGTCGAGGAGCTGCTGATCGGC
GGTGAGATGATTAAGGCGGGTACGTTGACCTTGGAGGAGGTGCGTCGTAAGTTTAACAATGGCGAAATCA
ACTTCTCGGGCGGCTCTTCTGGCGGCTCTTCCGGTTCCGAAACCCCAGGCACCAGCGAGAGCGCCACCCC
GGAGAGTTCTGGTGGCAGCTCTGGCGGCTCCTCTATGGAAGCGTACATTACGGAGATGGTTAGCCGTGAA
CGTGCGAACGAATTAGAAGTGTACGTGTACGTCTTTCCGCGTAAACAAAGCGACAACAACTATGAAGGTG
TTTACCACATTATGCGTGCTTGGCAGCGTGCGAATGATTTGCCACTGGCCTACAATCAGCATACCATCAT
GGCGTTCAGCCCGGTTCGTCACATGTGCGGTTATACCCCGATGGAGACCCAGAAGAGACACATCAACATT
GACAGCCCGTTTGAGCGTGCGCTGCTGGAGCGCCTGATTAAGAACTCCCTGATTTTTACTGCGGAACGCC
ACCTGCACGCTAAACGCGTCGGTCATGCACTGCGTCTGAATCAAGTTCAACAAATTCGTCAGGTTATCAT
TTACGAGGCCATCGAGTTATATGTAAATATTATTGAGAACCGCATTTCGATCGGCTTCCACCTGACCCAC
CAATTCGAGTACGTTTACACCCTGCAAAGCATGATCGAGCAGGGTAAAACCATCCGTCCGGGTATGCGTG
TCGTGCATAGCAACGGTCGTCAGCACTATACGTACACTGTGGAGAACGTTGCAACGTACGGCGTGACCGA
CCGCTGCCCGTTGTTGCAAACGAGCATCTACCAATATTATGTTGAGAAGGGCGCGCAGCACATCCTTCGC
ACTTTTACCCGTAGCACGCGTGTGATCCACGTGCGCACCAAAGAACAACGCCTCAGTTATGCTGCGACCC
TGTTGAAACCGCTTTGCACTTTCGAAACCATGCAACCGCAAGATGTTCTGAATGTGAGCAAGTGCATTAA
ACTGTCTGCAAGCAAGCGCATGAAATGTACCTATCGCTGGATTCAACAACTGCGGGCGCAATATCGTCAC
CTGACGTTCGCGCCGAACCCGTTCACCATCGCGCAGAATGGTTACAAACTGGATCAGCTGTCAACCCCGA
AGGTGCACTTCCACCGTGATTATGCGACTGTGGTGAGCGGGATGAAGACGGGTAAATTGTACAAGGGCGG
TAATATTAAAATTTCCGTGCTGTTTGACGAAGACTTCTATTTGAAGCACCACATCACCAAGAAGGACATC
TATCAATTCATTGCCGTACTGCAGAAAATCGCGATCGCGCAGGGCGTCAACATGACCATCAGCACCAGCA
CCAAGTCTATCACGGGTAAATTCACCGATGATTTTTTCCATCATTTTACGGAGGAGGTCGAAGCACTGCA
ACCTATCTTTGCCCAGACGACCGTGTTGGCGTTCATCACCAGCACTCACCTTTCCAACAAGAAGACCCGT
TCGTATCAGCTGCTGAAGCAGTACTTCGGTGGTAAATGGGACATCGCTTCGCAAGTTATCACTGAGAAGA
CCATTGAAGCGTTTCAGAAAATTCTGCATAAACACGGCCTAAAAAACTTCTACCCTAACGATGAACAACA
TTGTCTGCGTGTTATCGACGTTTTAAAAAACGAGTCATTTTACTACACAGTGATGAACATTTTGCTGGGT
GTTTACGTAAAAAGCGGCATCCAGCCGTGGATTTTAGCAAACACCACCCATTCCGACTGTTTTATCGGCA
TCGCGGTGAGCCATGAAAATGGCAACAGCGCTGCGGGTATGATGAACGTGATCGGTAGCCAAGGCCACCT
GATTCAGCAAGCACCGCTGAACGGAATCTTGGCTGGCGAAAAGATCGACGACACCCTTCTCGCGAATCTG
TTGAAGCAAATGATTAAGGCGTACCACACCCAATTTCAGCGCTTCCCGAAGCATATTACGATTCATCGTG
CCGGTTTTTGGCGTGAACATACCGCTTTGGTTGAAAAGATTATGAGCCATTATGAAATTACCTATGACAT
CGTGGAGATCATTAAGAAACCGAACAGACGTATGGCGTTCTTCAACTCGGTGGACAACACCTTTAGCACC
CGCCAGGGCACCGTTTACCAGCGTGGTAACGAGGCATTTCTGTGCGCTACCAATCCGCAACAAAAAGTAG
GCATGGCGCAGCCGATCAAGATCCACCAAGTGACCAAGACTCTGCCGTTCAGCCATATTATCGAGGATGT

TTACAACTTGAGCTTCTTACACATCCATGCCATGAACAAAATGCGCCTGCCGGCAACCATCCACTATGCT
GACCTGTCTGCGACGGCATACCAGCGAGGTCAGGTTATGCCGCGTAGCGGCAACCAGACCAATCTGCCAT
TCGTCtaagtcgacctcgagcaccaccaccaccaccactgagatccggctgctaacaaagcccgaaagga
agctgagttggctgctgccaccgctgagcaataactagcataaccccttggggcctctaaacgggtcttg
aggggttttttgctgaaaggaggaactatatccggat

The uppercase, underlined sequence encodes NLS-FokI-(d)KmAgo.

2) Target plasmids used in this study

A. Modified pMRS plasmid used to clone different γPNA target regions between the EcoRI and BamHI restriction sites (5,418 bp)

tagttattaatagtaatcaattacggggtcattagttcatagcccatatatggagttccgcgttacataa
cttacggtaaatggcccgcctggctgaccgcccaacgacccccgcccattgacgtcaataatgacgtatg
ttcccatagtaacgccaatagggactttccattgacgtcaatgggtggagtatttacggtaaactgccca
cttggcagtacatcaagtgtatcatatgccaagtacgccccctattgacgtcaatgacggtaaatggccc
gcctggcattatgcccagtacatgaccttatgggactttcctacttggcagtacatctacgtattagtca
tcgctattaccatggtgatgcggttttggcagtacatcaatgggcgtggatagcggtttgactcacgggg
atttccaagtctccaccccattgacgtcaatgggagtttgttttggcaccaaaatcaacgggactttcca
aaatgtcgtaacaactccgccccattgacgcaaatgggcggtaggcgtgtacggtgggaggtctatataa
gcagagctggtttagtgaaccgtcagatccgcttgccaccatggcctcctccgaggacgtcatcaaggag
ttcatgcgcttcaaggtgcgcatggagggctccgtgaacggccacgagttcgagatcgagggcgagggcg
agggccgcccctacgagggcacccagaccgccaagctgaaggtgaccaagggcggccccctgcccttcgc
ctgggacatcctgtcccctcagttccagtacggctccaaggcctacgtgaagcaccccgccgacatcccc
gactacttgaagctgtccttccccgagggcttcaagtgggagcgcgtgatgaacttcgaggacggcggcg
tggtgaccgtgacccaggactcctccctgcaggacggcgagttcatctacaaggtgaagctgcgcggcac
caacttcccctccgacggccccgtaatgcagaagaagaccatgggctgggaggcctccaccgagcggatg
taccccgaggacggcgccctgaagggcgagatcaagatgaggctgaagctgaaggacggcggccactacg
acgccgaggtcaagaccacctacatggccaagaagcccgtgcagctgcccggcgcctacaagaccgacat
caagctggacatcacctcccacaacgaggactacaccatcgtggaacagtacgagcgcgccgagggccgc
cactccaccggcgccgaattcCCGCTAGTGCGATGGCCTCATGGAAGCTTGATATCCAGCCAGGACAATT
TACCgatCCATGAGGCCATCGCACTAGGGGggatccagtgagcaagggcgaggagctgttcaccggggtg
gtgcccatcctggtcgagctggacggcgacgtaaacggccacaagttcagcgtgtccggcgagggcgagg
gcgatgccacctacggcaagctgaccctgaagttcatctgcaccaccggcaagctgcccgtgccctggcc
TacActGgtTacTacATtAacAtaTggAgtCcaAtgTttTagccgctaccccgaccacatgaagcagcac
gacttcttcaagtccgccatgcccgaaggctacgtccaggagcgcaccatcttcttcaaggacgacggca
actacaagacccgcgccgaggtgaagttcgagggcgacaccctggtgaaccgcatcgagctgaagggcat
cgacttcaaggaggacggcaacatcctggggcacaagctggagtacaactacaacagccacaacgtctat
atcatggccgacaagcagaagaacggcatcaaggtgaacttcaagatccgccacaacatcgaggacggca
gcgtgcagctcgccgaccactaccagcagaacacccccatcggcgacggccccgtgctgctgcccgacaa
ccactacctgagcacccagtccgccctgagcaaagaccccaacgagaagcgcgatcacatggtcctgctg
gagttcgtgaccgccgccgggatcactctcggcatggacgagctgtacaagtaagcggccgcgactctag
atcataatcagccataccacatttgtagaggttttacttgctttaaaaaacctcccacacctccccctga
acctgaaacataaaatgaatgcaattgttgttgttaacttgtttattgcagcttataatggttacaaata
aagcaatagcatcacaaatttcacaaataaagcatttttttcactgcattctagttgtggtttgtccaaa
ctcatcaatgtatcttaaggcgtaaattgtaagcgttaatattttgttaaaattcgcgttaaatttttgt
taaatcagctcattttttaaccaataggccgaaatcggcaaaatcccttataaatcaaaagaatagaccg
agatagggttgagtgttgttccagtttggaacaagagtccactattaaagaacgtggactccaacgtcaa
agggcgaaaaaccgtctatcagggcgatggcccactacgtgaaccatcaccctaatcaagttttttgggg

tcgaggtgccgtaaagcactaaatcggaaccctaaagggagcccccgatttagagcttgacggggaaagc
cggcgaacgtggcgagaaaggaagggaagaaagcgaaaggagcgggcgctagggcgctggcaagtgtagc
ggtcacgctgcgcgtaaccaccacacccgccgcgcttaatgcgccgctacagggcgcgtcaggtggcact
tttcggggaaatgtgcgcggaacccctatttgtttatttttctaaatacattcaaatatgtatccgctca
tgagacaataaccctgataaatgcttcaataatattgaaaaaggaagagtcctgaggcggaaagaaccag
ctgtggaatgtgtgtcagttagggtgtggaaagtccccaggctccccagcaggcagaagtatgcaaagca
tgcatctcaattagtcagcaaccaggtgtggaaagtccccaggctccccagcaggcagaagtatgcaaag
catgcatctcaattagtcagcaaccatagtcccgcccctaactccgcccatcccgcccctaactccgccc
agttccgcccattctccgccccatggctgactaattttttttatttatgcagaggccgaggccgcctcgg
cctctgagctattccagaagtagtgaggaggcttttttggaggcctaggcttttgcaaagatcgatcaag
agacaggatgaggatcgtttcgcatgattgaacaagatggattgcacgcaggttctccggccgcttgggt
ggagaggctattcggctatgactgggcacaacagacaatcggctgctctgatgccgccgtgttccggctg
tcagcgcaggggcgcccggttctttttgtcaagaccgacctgtccggtgccctgaatgaactgcaagacg
aggcagcgcggctatcgtggctggccacgacgggcgttccttgcgcagctgtgctcgacgttgtcactga
agcgggaagggactggctgctattgggcgaagtgccggggcaggatctcctgtcatctcaccttgctcct
gccgagaaagtatccatcatggctgatgcaatgcggcggctgcatacgcttgatccggctacctgcccat
tcgaccaccaagcgaaacatcgcatcgagcgagcacgtactcggatggaagccggtcttgtcgatcagga
tgatctggacgaagagcatcaggggctcgcgccagccgaactgttcgccaggctcaaggcgagcatgccc
gacggcgaggatctcgtcgtgacccatggcgatgcctgcttgccgaatatcatggtggaaaatggccgct
tttctggattcatcgactgtggccggctgggtgtggcggaccgctatcaggacatagcgttggctacccg
tgatattgctgaagagcttggcggcgaatgggctgaccgcttcctcgtgctttacggtatcgccgctccc
gattcgcagcgcatcgccttctatcgccttcttgacgagttcttctgagcgggactctggggttcgaaat
gaccgaccaagcgacgcccaacctgccatcacgagatttcgattccaccgccgccttctatgaaaggttg
ggcttcggaatcgttttccgggacgccggctggatgatcctccagcgcggggatctcatgctggagttct
tcgcccaccctagggggaggctaactgaaacacggaaggagacaataccggaaggaacccgcgctatgac
ggcaataaaaagacagaataaaacgcacggtgttgggtcgtttgttcataaacgcggggttcggtcccag
ggctggcactctgtcgataccccaccgagaccccattggggccaatacgcccgcgtttcttccttttccc
caccccaccccccaagttcgggtgaaggcccagggctcgcagccaacgtcggggcggcaggccctgccat
agcctcaggttactcatatatactttagattgatttaaaacttcatttttaatttaaaaggatctaggtg
aagatcctttttgataatctcatgaccaaaatcccttaacgtgagttttcgttccactgagcgtcagacc
ccgtagaaaagatcaaaggatcttcttgagatcctttttttctgcgcgtaatctgctgcttgcaaacaaa
aaaaccaccgctaccagcggtggtttgtttgccggatcaagagctaccaactctttttccgaaggtaact
ggcttcagcagagcgcagataccaaatactgtccttctagtgtagccgtagttaggccaccacttcaaga
actctgtagcaccgcctacatacctcgctctgctaatcctgttaccagtggctgctgccagtggcgataa
gtcgtgtcttaccgggttggactcaagacgatagttaccggataaggcgcagcggtcgggctgaacgggg
ggttcgtgcacacagcccagcttggagcgaacgacctacaccgaactgagatacctacagcgtgagctat
gagaaagcgccacgcttcccgaagggagaaaggcggacaggtatccggtaagcggcagggtcggaacagg
agagcgcacgagggagcttccagggggaaacgcctggtatctttatagtcctgtcgggtttcgccacctc
tgacttgagcgtcgatttttgtgatgctcgtcaggggggcggagcctatggaaaaacgccagcaacgcgg
cctttttacggttcctggccttttgctggccttttgctcacatgttctttcctgcgttatcccctgattc
tgtggataaccgtattaccgccatgcat

B. pUC19 plasmid used to clone all γPNA target regions between EcoRI and BamHI restriction sites (2,686 bp)

tcgcgcgtttcggtgatgacggtgaaaacctctgacacatgcagctcccggagacggtcacagcttgtct
gtaagcggatgccgggagcagacaagcccgtcagggcgcgtcagcgggtgttggcgggtgtcggggctgg
cttaactatgcggcatcagagcagattgtactgagagtgcaccatatgcggtgtgaaataccgcacagat
gcgtaaggagaaaataccgcatcaggcgccattcgccattcaggctgcgcaactgttgggaagggcgatc
ggtgcgggcctcttcgctattacgccagctggcgaaagggggatgtgctgcaaggcgattaagttgggta

```
acgccagggttttcccagtcacgacgttgtaaaacgacggccagtgaattcGAGCTCGGTACCCGGggat
cctctagagtcgacctgcaggcatgcaagcttggcgtaatcatggtcatagctgtttcctgtgtgaaatt
gttatccgctcacaattccacacaacatacgagccggaagcataaagtgtaaagcctggggtgcctaatg
agtgagctaactcacattaattgcgttgcgctcactgcccgctttccagtcgggaaacctgtcgtgccag
ctgcattaatgaatcggccaacgcgcggggagaggcggtttgcgtattgggcgctcttccgcttcctcgc
tcactgactcgctgcgctcggtcgttcggctgcggcgagcggtatcagctcactcaaaggcggtaatacg
gttatccacagaatcaggggataacgcaggaaagaacatgtgagcaaaaggccagcaaaaggccaggaac
cgtaaaaaggccgcgttgctggcgtttttccataggctccgcccccctgacgagcatcacaaaaatcgac
gctcaagtcagaggtggcgaaacccgacaggactataaagataccaggcgtttccccctggaagctccct
cgtgcgctctcctgttccgaccctgccgcttaccggatacctgtccgcctttctcccttcgggaagcgtg
gcgctttctcatagctcacgctgtaggtatctcagttcggtgtaggtcgttcgctccaagctgggctgtg
tgcacgaaccccccgttcagcccgaccgctgcgccttatccggtaactatcgtcttgagtccaacccggt
aagacacgacttatcgccactggcagcagccactggtaacaggattagcagagcgaggtatgtaggcggt
gctacagagttcttgaagtggtggcctaactacggctacactagaagaacagtatttggtatctgcgctc
tgctgaagccagttaccttcggaaaaagagttggtagctcttgatccggcaaacaaaccaccgctggtag
cggtggtttttttgtttgcaagcagcagattacgcgcagaaaaaaaggatctcaagaagatcctttgatc
ttttctacggggtctgacgctcagtggaacgaaaactcacgttaagggattttggtcatgagattatcaa
aaaggatcttcacctagatccttttaaattaaaaatgaagttttaaatcaatctaaagtatatatgagta
aacttggtctgacagttaccaatgcttaatcagtgaggcacctatctcagcgatctgtctatttcgttca
tccatagttgcctgactccccgtcgtgtagataactacgatacgggagggcttaccatctggccccagtg
ctgcaatgataccgcgagacccacgctcaccggctccagatttatcagcaataaaccagccagccggaag
ggccgagcgcagaagtggtcctgcaactttatccgcctccatccagtctattaattgttgccgggaagct
agagtaagtagttcgccagttaatagtttgcgcaacgttgttgccattgctacaggcatcgtggtgtcac
gctcgtcgtttggtatggcttcattcagctccggttcccaacgatcaaggcgagttacatgatcccccat
gttgtgcaaaaaagcggttagctccttcggtcctccgatcgttgtcagaagtaagttggccgcagtgtta
tcactcatggttatggcagcactgcataattctcttactgtcatgccatccgtaagatgcttttctgtga
ctggtgagtactcaaccaagtcattctgagaatagtgtatgcggcgaccgagttgctcttgcccggcgtc
aatacgggataataccgcgccacatagcagaactttaaaagtgctcatcattggaaaacgttcttcgggg
cgaaaactctcaaggatcttaccgctgttgagatccagttcgatgtaacccactcgtgcacccaactgat
cttcagcatcttttactttcaccagcgtttctgggtgagcaaaaacaggaaggcaaaatgccgcaaaaaa
gggaataagggcgacacggaaatgttgaatactcatactcttcctttttcaatattattgaagcatttat
cagggttattgtctcatgagcggatacatatttgaatgtatttagaaaaataaacaaataggggttccgc
gcacatttccccgaaaagtgccacctgacgtctaagaaaccattattatcatgacattaacctataaaaa
taggcgtatcacgaggccctttcgtc
```

Note: The *Eco*RI and *Bam*HI restriction sites are underlined and highlighted in yellow, respectively

Supplementary tables

Supplementary Table S1. Sequences of proteins used in this study

Protein name	Sequence
NLS-FokI-(d)CbAgo	SMAPKKKRKVGIHRGVPGGSMGSQLVKSELEEKKSELRHKLKYVPHEYIELIEIARNSTQD<br>RILEMKVMEFFMKVYGYRGKHLGGSRKPDGAIYTVGSPIDYGVIVDTKAYSGGYNLPIGQA<br>DEMQRYVEENQTRNKHINPNEWWKVYPSSVTEFKFLFVSGHFKGNYKAQLTRLNHITNCNG<br>AVLSVEELLIGGEMIKAGTLTLEEVRRKFNNGEINFSGGSSGGSSGSETPGTSESATPESS<br>GGSSGGSSMNNLTFEAFEGIGQLNELNFYKYRLIGKGQIDNVHQAIWSVKYKLQANNFFKP<br>VFVKGEILYSLDELKVIPEFENVEVILDGNIILSISENTDIYKDVIVFYINNALKNIKDIT<br>NYRKYITKNTDEIICKSILTTNLKYQYMKSEKGFKLQRKFKISPVVFRNGKVILYLNCSSD<br>FSTDKSIYEMLNNGLDVVGLQVKNRWTNSNGNIFIEEVLDKSISEPGTSGKLGQSLIDYYI<br>NGNQKYRVEKFTDEDKKAKVIKAKIKNKTYNYIPQALTPVITREYLSHTDKKFSKQIENVI<br>KMDMNYRYQTLKSFVEDIGVIKELNNLHFKNQYYTNFDFMGFESGILEEPVLMGANGKIKD<br>KKQIFINGFFKNPKENVKFGVLYPEGCMENAQSIARSILDFATAGKYNKQENKYISKNLMN<br>IGFKPSECIFESYKLGDITEYKATARKLKEHEKVGFVIAVIPDMNESEVENPYNPFKKVWA<br>KLNIPSQMITLKTTEKFKNIVDKSGLYYLHNIALNILGKIGGIPWIIKDMPGNIDCFIGLA<br>VGTREKGIHFPACSVLFDKYGKLINYYKPTIPQSGEKIAETILQEIFDNVLISYKEENGEY<br>PKNIVIHRAGFSRENIDWYKEYFDKKGIKFNIIEVKKNIPVKIAKVVGSNICNPIKGSYVL<br>KNDKAFIVTTDIKDGVASPNPLKIEKTYGDVEMKSILEQIYSLSQIHVGSTKSLRLPITTG<br>YADKICKAIEYIPQGVVDNRLFFL
NLS-FokI-(d)KmAgo	PKKKRKVGIHRGVPGGSMGSQLVKSELEEKKSELRHKLKYVPHEYIELIEIARNSTQDRIL<br>EMKVMEFFMKVYGYRGKHLGGSRKPDGAIYTVGSPIDYGVIVDTKAYSGGYNLPIGQADEM<br>QRYVEENQTRNKHINPNEWWKVYPSSVTEFKFLFVSGHFKGNYKAQLTRLNHITNCNGAVL<br>SVEELLIGGEMIKAGTLTLEEVRRKFNNGEINFSGGSSGGSSGSETPGTSESATPESSGGS<br>SGGSSMEAYITEMVSRERANELEVYVYVFPRKQSDNNYEGVYHIMRAWQRANDLPLAYNQH<br>TIMAFSPVRHMCGYTPMETQKRHINIDSPFERALLERLIKNSLIFTAERHLHAKRVGHALR<br>LNQVQQIRQVIIYEAIELYVNIIENRISIGFHLTHQFEYVYTLQSMIEQGKTIRPGMRVVH<br>SNGRQHYTYTVENVATYGVTDRCPLLQTSIYQYYVEKGAQHILRTFTRSTRVIHVRTKEQR<br>LSYAATLLKPLCTFETMQPQDVLNVSKCIKLSASKRMKCTYRWIQQLRAQYRHLTFAPNPF<br>TIAQNGYKLDQLSTPKVHFHRDYATVVSGMKTGKLYKGGNIKISVLFDEDFYLKHHITKKD<br>IYQFIAVLQKIAIAQGVNMTISTSTKSITGKFTDDFFHHFTEEVEALQPIFAQTTVLAFIT<br>STHLSNKKTRSYQLLKQYFGGKWDIASQVITEKTIEAFQKILHKHGLKNFYPNDEQHCLRV<br>IDVLKNESFYYTVMNILLGVYVKSGIQPWILANTTHSDCFIGIAVSHENGNSAAGMMNVIG<br>SQGHLIQQAPLNGILAGEKIDDTLLANLLKQMIKAYHTQFQRFPKHITIHRAGFWREHTAL<br>VEKIMSHYEITYDIVEIIKKPNRRMAFFNSVDNTFSTRQGTVYQRGNEAFLCATNPQQKVG<br>MAQPIKIHQVTKTLPFSHIIEDVYNLSFLHIHAMNKMRLPATIHYADLSATAYQRGQVMPR<br>SGNQTNLPFV

Note: the different colors indicate the SV40 NLS, FokI cleavage domain, (d)CbAgo, (d)KmAgo, the D- (in intact pAgo) to-A (in (d)pAgo) mutation in the DEDX motif.
Yellow highlighted sequence is a flexible linker.

Supplementary Table S2. PNAs (peptide nucleic acids) used in this study

Name	Sequence	PNA modifications
γPNA1	H-KKK-TCTTCTTCTGCTCGGACTCA-KKK-propargylglycine-NH_2	Gamma-alanine at all bases
γPNA2	H-KKK-GGCTCCCATCACATCAACC-KKK-propargylglycine-NH_2	Gamma-alanine at all bases
γPNA3	H-KKK-GCCCACCCTCGTGACCACCC-KKK-propargylglycine-NH_2	Gamma-alanine at all bases
γPNA4	H-KKK-TCCGCATTGAGAACCTCCCT-KKK-propargylglycine-NH_2	Gamma-alanine at all bases
γPNA5	H-KKK-AAACACTACATCTGCAATAT-KKK-propargylglycine-NH_2	Gamma-alanine at all bases
γPNA6	H-KKK-CAGGGTGGCTCTTCAGTGCA-KKK-propargylglycine-NH_2	Gamma-alanine at all bases
γPNA3-16nt	H-KKK-GCCCACCCTCGTGACC-KKK-propargylglycine-NH_2	Gamma-alanine at all bases
γPNA3-14nt	H-KKK-GCCCACCCTCGTGA-KKK-propargylglycine-NH_2	Gamma-alanine at all bases
γPNA3-10nt	H-KKK-GCCCACCCTC-KKK-propargylglycine-NH_2	Gamma-alanine at all bases
γPNA4-16nt	H-KKK-TCCGCATTGAGAACCT-KKK-propargylglycine-NH_2	Gamma-alanine at all bases
γPNA4-14nt	H-KKK-TCCGCATTGAGAAC-KKK-propargylglycine-NH_2	Gamma-alanine at all bases
γPNA4-10nt	H-KKK-TCCGCATTGA-KKK-propargylglycine-NH_2	Gamma-alanine at all bases

H, free amine at the N terminus.
NH_2, amide at the C terminus.
K, lysine.

Supplementary Table S3. Oligos used for cloning target regions into the modified pMRS plasmid

Name of the oligo	Sequence (5′ to 3′)	Figure(s) and supplementary figure(s)
γPNA1_γPNA2 6nt_IN_top	AATTATGAGTCCGAGCAGAAGAAGAGAGCTCGG CTCCCATCACATCAACCG	1C, 2B, 3A, 3B, 4A, 4B, 4C, 5, 6B, 8, 9B, S1C, S1D, S2A, S2B, S3, S4B, S5, S6, S7B, S7C, S8A, S8B
γPNA1_γPNA2 6nt_IN_bottom	GATCCGGTTGATGTGATGGGAGCCGAGCTCTCT TCTTCTGCTCGGACTCAT	
γPNA3_γPNA4 target_6nt_ _top	GATCCGCCCACCCTCGTGACCACCCGAGCTCAG GGAGGTTCTCAATGCGGAG	6A, 7B, 7C
γPNA3_γPNA4 target_6nt_ bottom	AATTCTCCGCATTGAGAACCTCCCTGAGCTCGG GTGGTCACGAGGGTGGGCG	
γPNA1_γPNA2 6nt_OUT_top	AATTATCTTCTTCTGCTCGGACTCAGAGCTCGG TTGATGTGATGGGAGCCA	S4B
γPNA1_γPNA2 6nt_OUT_bottom	GATCTGGCTCCCATCACATCAACCGAGCTCTGA GTCCGAGCAGAAGAAGAT	
γPNA1_γPNA2 6nt_SAME STRAND_top	AATTATGAGTCCGAGCAGAAGAAGAGAGCTCGG TTGATGTGATGGGAGCCG	S4B
γPNA1_γPNA2 6nt_SAME STRAND_bottom	GATCCGGCTCCCATCACATCAACCGAGCTCTCT TCTTCTGCTCGGACTCAT	
γPNA1_γPNA2 10nt_IN_top	AATTATGAGTCCGAGCAGAAGAAGATGGAGCTC TGGGCTCCCATCACATCAACCG	S4B
γPNA1_γPNA2 10nt_IN_bottom	GATCCGGTTGATGTGATGGGAGCCCAGAGCTCC ATCTTCTTCTGCTCGGACTCAT	
γPNA1_γPNA2 10nt_OUT_top	AATTACTCTTCTTCTGCTCGGACTCATGGAGCT CTCGGTTGATGTGATGGGAGCCG	S4B
γPNA1_γPNA2 10nt_OUT_bottom	GATCCGGCTCCCATCACATCAACCGAGAGCTCC ATGAGTCCGAGCAGAAGAAGAGT	
γPNA1_γPNA2 10nt_SAME STRAND_top	AATTATGAGTCCGAGCAGAAGAAGATGGAGCTC TGGGTTGATGTGATGGGAGCCG	S4B
γPNA1_γPNA2 10nt_SAME STRAND_bottom	GATCCGGCTCCCATCACATCAACCCAGAGCTCC ATCTTCTTCTGCTCGGACTCAT	
γPNA1_γPNA2 15nt_IN_top	AATTACTGAGTCCGAGCAGAAGAAGATGTGCGA GCTCACTGGGCTCCCATCACATCAACCG	S4B
γPNA1_γPNA2 15nt_IN_bottom	GATCCGGTTGATGTGATGGGAGCCCAGTGAGCT CGCACATCTTCTTCTGCTCGGACTCAGT	
γPNA1_γPNA2 15nt_OUT_top	AATTACTCTTCTTCTGCTCGGACTCATGTGCGA GCTCACTGGGTTGATGTGATGGGAGCCG	9A, S4B
γPNA1_γPNA2 15nt_OUT_bottom	GATCCGGCTCCCATCACATCAACCCAGTGAGCT CGCACATGAGTCCGAGCAGAAGAAGAGT	

γPNA1_γPNA2 15nt_SAME STRAND_top	AATTACTGAGTCCGAGCAGAAGAAGATGTGCGAGCTCACTGGGTTGATGTGATGGGAGCCG	S4B
γPNA1_γPNA2 15nt_SAME STRAND_bottom	GATCCGGCTCCCATCACATCAACCCAGTGAGCTCGCACATCTTCTTCTGCTCGGACTCAGT	
γPNA1_γPNA2 20nt_IN_top	AATTACTGAGTCCGAGCAGAAGAAGATGTGATCGAGCTCAGAACTGGGCTCCCATCACATCAACCG	S4B
γPNA1_γPNA2 20nt_IN_bottom	GATCCGGTTGATGTGATGGGAGCCCAGTTCTGAGCTCGATCACATCTTCTTCTGCTCGGACTCAGT	
γPNA1_γPNA2 20nt_OUT_top	AATTACTCTTCTTCTGCTCGGACTCATGTGATCGAGCTCAGAACTGGGTTGATGTGATGGGAGCCG	S4B
γPNA1_γPNA2 20nt_OUT_bottom	GATCCGGCTCCCATCACATCAACCCAGTTCTGAGCTCGATCACATGAGTCCGAGCAGAAGAAGAGT	
γPNA1_γPNA2 20nt_SAME STRAND_top	AATTACTGAGTCCGAGCAGAAGAAGATGTGATCGAGCTCAGAACTGGGTTGATGTGATGGGAGCCG	S4B
γPNA1_γPNA2 20nt_SAME STRAND_bottom	GATCCGGCTCCCATCACATCAACCCAGTTCTGAGCTCGATCACATCTTCTTCTGCTCGGACTCAGT	
γPNA1_γPNA2 25nt_IN_top	AATTACTGAGTCCGAGCAGAAGAAGATGTGATCGAGAGCTCGGTAGAACTGGGCTCCCATCACATCAACCG	S4B
γPNA1_γPNA2 25nt_IN_bottom	GATCCGGTTGATGTGATGGGAGCCCAGTTCTACCGAGCTCTCGATCACATCTTCTTCTGCTCGGACTCAGT	
γPNA1_γPNA2 25nt_OUT_top	AATTACTCTTCTTCTGCTCGGACTCATGTGATCGAGAGCTCGGTAGAACTGGGTTGATGTGATGGGAGCCG	S4B
γPNA1_γPNA2 25nt_OUT_bottom	GATCCGGCTCCCATCACATCAACCCAGTTCTACCGAGCTCTCGATCACATGAGTCCGAGCAGAAGAAGAGT	
γPNA1_γPNA2 25nt_SAME STRAND_top	AATTACTGAGTCCGAGCAGAAGAAGATGTGATCGAGAGCTCGGTAGAACTGGGTTGATGTGATGGGAGCCG	S4B
γPNA1_γPNA2 25nt_SAME STRAND_bottom	GATCCGGCTCCCATCACATCAACCCAGTTCTACCGAGCTCTCGATCACATCTTCTTCTGCTCGGACTCAGT	
γPNA1_γPNA2 30nt_IN_top	AATTACTGAGTCCGAGCAGAAGAAGATGTGCTCAGTCAGAGCTCTGATGAGAACTGGGCTCCCATCACATCAACCG	S4B
γPNA1_γPNA2 30nt_IN_bottom	GATCCGGTTGATGTGATGGGAGCCCAGTTCTCATCAGAGCTCTGACTGAGCACATCTTCTTCTGCTCGGACTCAGT	
γPNA1_γPNA2 30nt_OUT_top	aattATCTTCTTCTGCTCGGACTCATGTGCTCAGTCAGAGCTCTGATGAGAACTGGGTTGATGTGATGGGAGCCG	S4B
γPNA1_γPNA2 30nt_OUT_bottom	gatcCGGCTCCCATCACATCAACCCAGTTCTCATCAGAGCTCTGACTGAGCACATGAGTCCGAGCAGAAGAAGAT	

γPNA1_γPNA2 30nt_SAME STRAND_top	AATTACTGAGTCCGAGCAGAAGAAGATGTGCTCAGTCAGAGCTCTGATGAGAACTGGGTTGATGTGATGGGAGCCG	S4B
γPNA1_γPNA2 30nt_SAME STRAND_bottom	GATCCGGCTCCCATCACATCAACCCAGTTCTCATCAGAGCTCTGACTGAGCACATCTTCTTCTGCTCGGACTCAGT	
γPNA1_γPNA2 40nt_IN_top	AATTATGAGTCCGAGCAGAAGAAGATGTGCTCAGTCAAATCGAGCTCGATACTTGATGAGAACTGGGCTCCCATCACATCAACCG	S4B
γPNA1_γPNA2 40nt_IN_bottom	GATCCGGTTGATGTGATGGGAGCCCAGTTCTCATCAAGTATCGAGCTCGATTTGACTGAGCACATCTTCTTCTGCTCGGACTCAT	
γPNA1_γPNA2 40nt_OUT_top	AATTATCTTCTTCTGCTCGGACTCATGTGCTCAGTCAAATCGAGCTCGATACTTGATGAGAACTGGGTTGATGTGATGGGAGCCG	S4B
γPNA1_γPNA2 40nt_OUT_bottom	GATCCGGCTCCCATCACATCAACCCAGTTCTCATCAAGTATCGAGCTCGATTTGACTGAGCACATGAGTCCGAGCAGAAGAAGAT	
γPNA1_γPNA2 40nt_SAME STRAND_top	AATTATGAGTCCGAGCAGAAGAAGATGTGCTCAGTCAAATCGAGCTCGATACTTGATGAGAACTGGGTTGATGTGATGGGAGCCG	S4B
γPNA1_γPNA2 40nt_SAME STRAND_bottom	GATCCGGCTCCCATCACATCAACCCAGTTCTCATCAAGTATCGAGCTCGATTTGACTGAGCACATCTTCTTCTGCTCGGACTCAT	
γPNA1_γPNA2 50nt_IN_top	AATTATGAGTCCGAGCAGAAGAAGATGTGCTCAGTCAAATCGATCGAGAGCTCCATCGATACTTGATGAGAACTGGGCTCCCATCACATCAACCG	S4B
γPNA1_γPNA2 50nt_IN_bottom	GATCCGGTTGATGTGATGGGAGCCCAGTTCTCATCAAGTATCGATGGAGCTCTCGATCGATTTGACTGAGCACATCTTCTTCTGCTCGGACTCAT	
γPNA1_γPNA2 50nt_OUT_top	AATTATCTTCTTCTGCTCGGACTCATGTGCTCAGTCAAATCGATCGAGAGCTCCATCGATACTTGATGAGAACTGGGTTGATGTGATGGGAGCCG	S4B
γPNA1_γPNA2 50nt_OUT_bottom	GATCCGGCTCCCATCACATCAACCCAGTTCTCATCAAGTATCGATGGAGCTCTCGATCGATTTGACTGAGCACATGAGTCCGAGCAGAAGAAGAT	
γPNA1_γPNA2 50nt_SAME STRAND_top	AATTATGAGTCCGAGCAGAAGAAGATGTGCTCAGTCAAATCGATCGAGAGCTCCATCGATACTTGATGAGAACTGGGTTGATGTGATGGGAGCCG	S4B
γPNA1_γPNA2 50nt_SAME STRAND_bottom	GATCCGGCTCCCATCACATCAACCCAGTTCTCATCAAGTATCGATGGAGCTCTCGATCGATTTGACTGAGCACATCTTCTTCTGCTCGGACTCAT	
ssDNA target 1519	AATTAGCACTGCACGCCGTAGGTCTGGGTGGTCACGAGGGTGGGCATCCGCATTGAGAACCTCCCTAAGTCACAAAATCTGGATATAGTGAGCTC	S9

Supplementary Table S4. Sequencing primers used in this study

Primer number	Primer name	Sequence (5′ to 3′)	Used for
1442	RFP-Seq-F2	GGCCCCGTAATGCAGAAGAAG	Confirmation of cloning into pMRS vector and sanger sequencing
1444	GFP-Seq-R2	CTCGGCGCGGGTCTTGTAG	Confirmation of cloning into pMRS vector and sanger sequencing
1447	pUC19-F	GGGCTGGCTTAACTATGCGG	Confirmation of cloning into pUC19 vector
1448	pUC19-R	CGAGGAAGCGGAAGAGCG	Confirmation of cloning into pUC19 vector

Supplementary Table S5. Guide DNAs used in this study

Name	Designation	Sequence (5′ to 3′)
gDNA γPNA 1	1749	/5PHOS/TCCGAGCAGAAGAAGA
gDNA γPNA 2	1750	/5PHOS/TTGATGTGATGGGAGC
γPNA1 full PT gDNA	1921	/5PHOS/T*C*C*G*A*G*C*A*G*A*A*G*A*A*G*A
γPNA2 full PT gDNA	1922	/5PHOS/T*T*G*A*T*G*T*G*A*T*G*G*G*A*G*C
gRNA γPNA1_16nt	1753	/5PHOS/rUrCrCrGrArGrCrArGrArArGrArArGrA
gRNA γPNA2_16nt	1754	/5PHOS/rUrUrGrArUrGrUrGrArUrGrGrGrArGrC
gDNA γPNA3_16nt	1561	/5PHOS/TGGTCACGAGGGTGGG
gDNA γPNA 4_16nt	1545	/5PHOS/AGGTTCTCAATGCGGA
gDNA γPNA3_20-nt	1733	/5PHOS/GGGTGGTCACGAGGGTGGGC
gDNA γPNA3_18-nt	1734	/5PHOS/GTGGTCACGAGGGTGGGC
gDNA γPNA3_14-nt	1735	/5PHOS/TGGTCACGAGGGTG
gDNA γPNA3_12-nt	1736	/5PHOS/TGGTCACGAGGG
gDNA γPNA3_10-nt	1737	/5PHOS/TGGTCACGAG
gDNA γPNA 4_20-nt	1738	/5PHOS/AGGGAGGTTCTCAATGCGGA
gDNA γPNA 4_18-nt	1739	/5PHOS/GGAGGTTCTCAATGCGGA
gDNA γPNA 4_14-nt	1740	/5PHOS/AGGTTCTCAATGCG
gDNA γPNA 4_12-nt	1741	/5PHOS/AGGTTCTCAATG
gDNA γPNA 4_10-nt	1742	/5PHOS/AGGTTCTCAA
new 16-nt shorter guide 3	2144	/5PHOS/GGTCACGAGGGTGGGC
new 14-nt shorter guide 3	2145	/5PHOS/TCACGAGGGTGGGC
new 10-nt shorter guide 3	2146	/5PHOS/GAGGGTGGGC
new 14-nt shorter guide 4	2148	/5PHOS/GTTCTCAATGCGGA
new 10-nt shorter guide 4	2149	/5PHOS/TCAATGCGGA
1749_anchor_1nt	2414	/5PHOS/ACCGAGCAGAAGAAGA
1749_anchor_2nt	2415	/5PHOS/AGCGAGCAGAAGAAGA
1749_anchor_3nt	2416	/5PHOS/AGGGAGCAGAAGAAGA
1749_anchor_4nt	2417	/5PHOS/AGGCAGCAGAAGAAGA
1749_anchor_5nt	2418	/5PHOS/AGGCTGCAGAAGAAGA
1749_anchor_6nt	2419	/5PHOS/AGGCTCCAGAAGAAGA
1749_seed_1nt	2420	/5PHOS/TCCGAGCAGAAGAAGA
1749_seed_2nt	2421	/5PHOS/TCCGTGCAGAAGAAGA
1749_seed_3nt	2422	/5PHOS/TCCGTCCAGAAGAAGA
1749_seed_4nt	2423	/5PHOS/TCCGTCGAGAAGAAGA
1749_seed_5nt	2424	/5PHOS/TCCGTCGTGAAGAAGA
1749_seed_6nt	2425	/5PHOS/TCCGTCGTCAAGAAGA
1749_central_1nt	2426	/5PHOS/TCCGAGCACAAGAAGA
1749_central_2nt	2427	/5PHOS/TCCGAGCTCAAGAAGA
1749_central_3nt	2428	/5PHOS/TCCGAGCTCTAGAAGA
1749_central_4nt	2429	/5PHOS/TCCGAGGTCTAGAAGA
1749_central_5nt	2430	/5PHOS/TCCGAGGTCTTGAAGA
1749_central_6nt	2431	/5PHOS/TCCGACGTCTTGAAGA
1749_supplementary_1nt	2432	/5PHOS/TCCGAGCAGAAGTAGA

1749_supplementary_2nt	2433	/5PHOS/TCCGAGCAGAACTAGA
1749_supplementary_3nt	2434	/5PHOS/TCCGAGCAGATCTAGA
1749_supplementary_4nt	2435	/5PHOS/TCCGAGCAGTTCTAGA
1749_supplementary_5nt	2436	/5PHOS/TCCGAGCACTTCTAGA
1749_supplementary_6nt	2437	/5PHOS/TCCGAGCTCTTCTAGA
1749_tail_1nt	2438	/5PHOS/TCCGAGCAGAAGAAGT
1749_tail_2nt	2439	/5PHOS/TCCGAGCAGAAGAACT
1749_tail_3nt	2440	/5PHOS/TCCGAGCAGAAGATCT
1749_tail_4nt	2441	/5PHOS/TCCGAGCAGAAGTTCT
1749_tail_5nt	2442	/5PHOS/TCCGAGCAGAACTTCT
1749_tail_6nt	2443	/5PHOS/TCCGAGCAGATCTTCT
1749_Full mismatch guide	2444	/5PHOS/AGGCTCGTCTTCTTCT
1750_anchor_1nt	2446	/5PHOS/ATGATGTGATGGGAGC
1750_anchor_2nt	2447	/5PHOS/AAGATGTGATGGGAGC
1750_anchor_3nt	2448	/5PHOS/AACATGTGATGGGAGC
1750_anchor_4nt	2449	/5PHOS/AACTTGTGATGGGAGC
1750_anchor_5nt	2450	/5PHOS/AACTAGTGATGGGAGC
1750_anchor_6nt	2451	/5PHOS/AACTACTGATGGGAGC
1750_seed_1nt	2452	/5PHOS/TTGTTGTGATGGGAGC
1750_seed_2nt	2453	/5PHOS/TTGTAGTGATGGGAGC
1750_seed_3nt	2454	/5PHOS/TTGTACTGATGGGAGC
1750_seed_4nt	2455	/5PHOS/TTGTACAGATGGGAGC
1750_seed_5nt	2456	/5PHOS/TTGTACACATGGGAGC
1750_seed_6nt	2457	/5PHOS/TTGTACACTTGGGAGC
1750_central_1nt	2458	/5PHOS/TTGATGTGTTGGGAGC
1750_central_2nt	2459	/5PHOS/TTGATGTCTTGGGAGC
1750_central_3nt	2460	/5PHOS/TTGATGTCTAGGGAGC
1750_central_4nt	2461	/5PHOS/TTGATGACTAGGGAGC
1750_central_5nt	2462	/5PHOS/TTGATGACTACGGAGC
1750_central_6nt	2463	/5PHOS/TTGATCACTACGGAGC
1750_supplementary_1nt	2464	/5PHOS/TTGATGTGATGGCAGC
1750_supplementary_2nt	2465	/5PHOS/TTGATGTGATGCCAGC
1750_supplementary_3nt	2466	/5PHOS/TTGATGTGATCCCAGC
1750_supplementary_4nt	2467	/5PHOS/TTGATGTGAACCCAGC
1750_supplementary_5nt	2468	/5PHOS/TTGATGTGTACCCAGC
1750_supplementary_6nt	2469	/5PHOS/TTGATGTCTACCCAGC
1750_tail_1nt	2470	/5PHOS/TTGATGTGATGGGAGG
1750_tail_2nt	2471	/5PHOS/TTGATGTGATGGGACG
1750_tail_3nt	2472	/5PHOS/TTGATGTGATGGGTCG
1750_tail_4nt	2473	/5PHOS/TTGATGTGATGGCTCG
1750_tail_5nt	2474	/5PHOS/TTGATGTGATGCCTCG
1750_tail_6nt	2475	/5PHOS/TTGATGTGATCCCTCG
1750_Full mismatch guide	2476	/5PHOS/AACTACACTACCCTCG
gDNA 13% targeting bottom	1531	/5PHOS/TATTAATAGTAATCAA
gDNA 13% targeting top	1532	/5PHOS/TTGATTACTATTAATA
gDNA p22% targeting bottom	1533	/5PHOS/TAATAGTAATCAATTACG
gDNA 22% targeting top	1534	/5PHOS/CGTAATTGATTACTATTA
gDNA 27% targeting bottom	1535	/5PHOS/AATAATGACGTATGT

gDNA 27% targeting top	1536	/5PHOS/ACATACGTCATTATT
gDNA 31% targeting bottom	1537	/5PHOS/TATTAGTCATCGCTAT
gDNA 31% targeting top	1538	/5PHOS/ATAGCGATGACTAATA
gDNA 35% targeting bottom	1539	/5PHOS/TTGTTTTGGCACCAAAA
gDNA 35% targeting top	1540	/5PHOS/TTTTGGTGCCAAAACAA
gDNA 44% targeting top	1962	/5PHOS/TCAGTGGAACGAAAAC
gDNA 44% targeting bottom	1963	/5PHOS/GTTTTCGTTCCACTGA
gDNA 63% targeting top	1918	5/PHOS/AATGGGCGGTAGGCGT
gDNA 63% targeting bottom	1919	5/PHOS/ACGCCTACCGCCCATT

5/PHOS/, 5′-phosphorylation
N*, phosphorothioated nucleotide, N denotes for A/T/C/G
rN, RNA nucleotide, N denotes for A/U/C/G

Supplementary methods

***In vitro* FokI-(d)pAgo-mediated cleavage assay with circular or linear plasmid DNA**

1) γPNA invasion of target dsDNA (linear or circular)

- Prepare 1 μM stock of γPNA and aliquot.
- Recommended: prior to use heat the aliquots at 90°C for 20 min to reconstitute γPNA.

Component	Stock concentration	Volume (μL)	Final concentration
H_2O	–	2	–
MOPS buffer	10X	1	1X
γPNA 1	1 μM	1	100 nM
γPNA 3	1 μM	1	100 nM
Plasmid DNA	40 ng/μL (~ 3nM)	5	20 ng/μL (~ 1.5 nM)
		**10**	

Prepare at room temperature and incubate the reaction at 37°C for 1 h (for circular plasmids) or 5 h (for linear plasmids); lid temperature 39°C, no cooling step.

2.) Guide loading

- Prepare 200 nM FokI-(d)pAgo (FokI-(d)CbAgo or FokI-(d)KmAgo) protein secondary stock from primary protein stock using 1X NEB rCutSmart buffer.
- Assemble each reaction for paired FokI-(d)pAgos in two half-reactions.

Half reaction 1*:

Component	Stock concentration	Volume (μL)	Final concentration
H_2O	–	9	–
NEB rCutSmart buffer	10X	2	1X
Guide DNA1	1 μM	1	50 nM
Guide DNA2	1 μM	/	50 nM
NaCl	500 mM	2	50 mM
FokI-(d)pAgo	200 nM	2	20 nM
		16	

Half reaction 2*:

Component	Stock concentration	Volume (µL)	Final concentration
H_2O	–	9	–
NEB rCutSmart buffer	10X	2	1X
Guide DNA1	1 µM	/	50 nM
Guide DNA2	1 µM	1	50 nM
NaCl	500 mM	2	50 mM
FokI-(d)pAgo	200 nM	2	20 nM

*The above reactions are examples, using 20 nM FokI-(d)pAgo and 50 nM NaCl.

- Incubate the reactions at 37°C for 15 min; lid temperature 39°C, no cooling step.
- Combine 8 µL of half-reaction 1 with 8 µL of half-reaction 2 in one tube.
- Add 4 µL of invaded plasmid (80 ng final) or 2 µL of non-invaded plasmid (from 40 ng/µL stock) and 2 µL H_2O.
- Incubate the reaction at 37°C for 30 min; lid temperature 39°C.
- In case of a circular plasmid, add 1 µL of the appropriate restriction enzyme into the reaction and incubate at 37°C for 30 min. This step is not necessary for linearized targets.
- Add 1 µL of proteinase K (Invitrogen; catalog #: 25530049) and incubate at 37°C for 30 min.
- Add 4 µL of 6X Gel Loading Dye, Purple (NEB; catalog #: B7024S) and load the samples onto a 0.9% (w/v) agarose gel with GelRed® and run the 200-mL gel for 1 h at 145 V for 30 min.
